# The Core Protein of Classical Swine Fever Virus Is Dispensable for Virus Propagation In Vitro

**DOI:** 10.1371/journal.ppat.1002598

**Published:** 2012-03-22

**Authors:** Christiane Riedel, Benjamin Lamp, Manuela Heimann, Matthias König, Sandra Blome, Volker Moennig, Christian Schüttler, Heinz-Jürgen Thiel, Tillmann Rümenapf

**Affiliations:** 1 Institute of Virology, Faculty of Veterinary Medicine, Justus-Liebig Universität, Giessen, Germany; 2 Institute of Virology, Stiftung Tierärztliche Hochschule Hannover, Hannover, Germany; 3 Institute of Virology, Faculty of Medicine, Justus-Liebig Universität, Giessen, Germany; The Rockefeller University, United States of America

## Abstract

Core protein of *Flaviviridae* is regarded as essential factor for nucleocapsid formation. Yet, core protein is not encoded by all isolates (GBV- A and GBV- C). Pestiviruses are a genus within the family *Flaviviridae* that affect cloven-hoofed animals, causing economically important diseases like classical swine fever (CSF) and bovine viral diarrhea (BVD). Recent findings describe the ability of NS3 of classical swine fever virus (CSFV) to compensate for disabling size increase of core protein (Riedel et al., 2010). NS3 is a nonstructural protein possessing protease, helicase and NTPase activity and a key player in virus replication. A role of NS3 in particle morphogenesis has also been described for other members of the *Flaviviridae* (Patkar et al., 2008; Ma et al., 2008). These findings raise questions about the necessity and function of core protein and the role of NS3 in particle assembly. A reverse genetic system for CSFV was employed to generate poorly growing CSFVs by modification of the core gene. After passaging, rescued viruses had acquired single amino acid substitutions (SAAS) within NS3 helicase subdomain 3. Upon introduction of these SAAS in a nonviable CSFV with deletion of almost the entire core gene (Vp447_Δc_), virus could be rescued. Further characterization of this virus with regard to its physical properties, morphology and behavior in cell culture did not reveal major differences between wildtype (Vp447) and Vp447_Δc_. Upon infection of the natural host, Vp447_Δc_ was attenuated. Hence we conclude that core protein is not essential for particle assembly of a core-encoding member of the *Flaviviridae*, but important for its virulence. This raises questions about capsid structure and necessity, the role of NS3 in particle assembly and the function of core protein in general.

## Author Summary

Virus particles of members of the *Flaviviridae* consist of an inner complex of viral RNA genome and core protein that together form the nucleocapsid, and an outer lipid layer containing the viral glycoproteins. Functional analyses of core protein of the classical swine fever virus (CSFV), a pestivirus related to hepatitis C virus (HCV), led to the observation that crippling mutations or even complete deletion of the core gene were compensated by single amino acid substitutions in the helicase domain of non-structural protein 3 (NS3). NS3 is well conserved among the *Flaviviridae* and acts as protease and helicase. In addition to its essential role in RNA replication, NS3 apparently organizes the incorporation of RNA into budding virus particles. Characterization of core deficient CSFV particles (Vp447_Δc_) revealed that the lack of core had no effect with regard to thermostability, size, density, and morphology. Vp447_Δc_ was fully attenuated in the natural host. Our results provide evidence that core protein is not essential for virus assembly. Hence, Vp447_Δc_ might help to explain the enigmatic existence of GB viruses -A and -C, close relatives of HCV that do not encode an apparent core protein.

## Introduction

The genus pestivirus, together with the genera hepacivirus, flavivirus and the newly proposed genus pegivirus [1], constitutes the family *Flaviviridae*. Cloven-hoofed animals are affected by pestiviruses, which cause severe diseases like classical swine fever (CSF) and bovine viral diarrhea (BVD). Pestiviruses possess a single stranded RNA genome of positive polarity with one open reading frame (orf) encoding approximately 4000 amino acids (aa). The resulting polyprotein is processed co- and posttranslationally into at least 12 viral proteins by three viral and two cellular proteases [2].

Pestiviral particles are enveloped and contain three virus-encoded glycoproteins, E^rns^, E1 and E2. E^rns^ is unique for pestiviruses and is the only known viral structural protein with an uridinylate specific RNase domain belonging to the T2 RNase family [3], [4]. E1 and E2 or analogous proteins (prM, E) are encoded by all members of the *Flaviviridae*. Inside the virus particle, the viral genome is accompanied by a core protein. However, members of the proposed genus pegivirus, GBV- A and GBV- C [reviewed by 1], do not appear to encode a core protein. Pestiviruses encode a small, basic core protein, which, in contrast to hepaci- and flaviviruses, does not possess any predicted regular secondary structure and is intrinsically disordered [5], [6]. The pestiviral core protein has RNA chaperone activity [6] and its implicated functions are condensation of the viral RNA genome and subsequent packaging into virions. Its ability to bind RNA relies on the overall protein charge, which results in an unspecific affinity for nucleic acids [5]. The pestiviral core protein is processed at its N-terminus by the autoprotease N^pro^ [7], whereas the C-terminus is generated by signal peptide peptidase (SPP) cleavage [8]. Recent findings revealed that deletion of basic areas of classical swine fever virus (CSFV) core protein (aa 213–231 of the viral polyprotein) results in a ten-fold reduction of virus output, whereas deletion of small, less charged stretches (aa 194–198 and aa 208–212) leads to a more than 1000-fold drop in virus output [9]. This implicates a more complex mechanism of core function in particle morphogenesis, which is not solely relying on overall protein charge. Duplication and triplication of the CSFV core protein gene as well as integration of up to 3 yellow fluorescent protein (YFP) genes between 2 core coding regions yielded replication competent viruses whose virus output was approximately 100-fold reduced in comparison to wildtype, revealing a high tolerance of core protein to size increase. We also reported the rescue of a CSFV encoding an YFP-core fusion protein by a single amino acid substitution in the NS3 helicase domain (N2256Y) [9]. This finding points to an ability of NS3 to substitute core functions.

For all members of the *Flaviviridae*, there is increasing evidence that nonstructural proteins are required for virus morphogenesis [reviewed by 10]. Single amino acid residues in the NS3 helicase domain of yellow fever virus (YFV) and hepatitis C virus (HCV) have been described as important for particle formation [11], [12]. Apart from NS3, p7, NS2 and NS5A have been reported as factors involved in HCV particle generation [13]–[17].

The pestiviral NS3 is a multifunctional molecule possessing protease, NTPase and helicase activity [18]–[22] and shares similarity with the analogous protein of hepaci- and flaviviruses. Its uncleaved precursor, NS2–3, has been reported to be essential for particle formation [23], [24].

In the present study, we describe the ability of CSFV NS3 to compensate for functionally compromised core proteins and even the deletion of nearly 90% of the core gene by acquisition of single codon rescue mutations in its helicase subdomain 3. These findings provide strong evidence for a major role of the NS3 helicase domain in pestiviral particle assembly and implicate questions about the function of core protein. As members of the newly proposed genus pegivirus [1] – namely GBV- A and GBV- C - do not encode an obvious core protein, we provide experimental evidence that loss of the core coding region is tolerated by another member of the *Flaviviridae*.

## Results

### Single amino acid substitutions in the NS3 helicase domain rescue CSFVs encoding modified Core proteins

**Figure 1:**
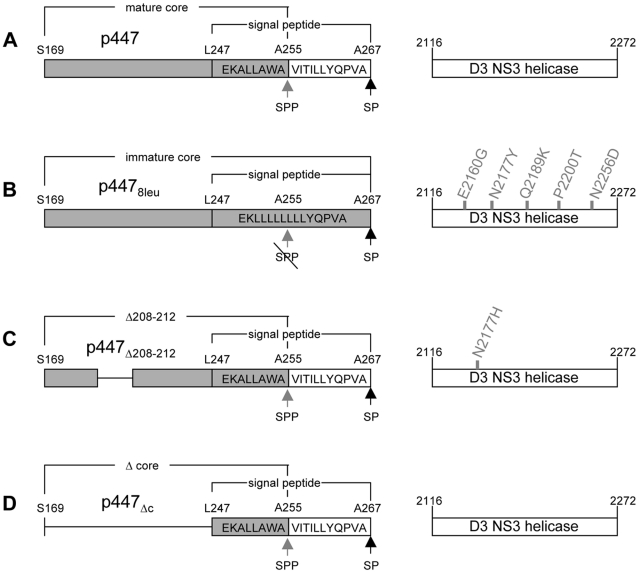
Illustration of modifications introduced into the core protein of CSFV and spontaneous occurence of rescue mutations in NS3 helicase domain 3. Depicted are the core regions of CSFV constructs (A) p447, (B) p447_8leu_, (C) p447_Δ208–212_, (D) p447_Δc_ starting with Serine 169 at the N-terminus of core protein, and ending with Alanine 267 at the signal-peptidase cleavage site. Deleted amino acids are represented by a black line. The signal peptide and its constituting amino acids are indicated. Gray background represents expressed protein. The putative NS3 helicase subdomain 3 is indicated as bar starting with amino acid 2116 of the polyprotein and ending with amino acid 2272. Spontaneously occurring rescue mutations are indicated for the respective core modifications, where the amino acid before the number of the residue represents the original residue and the amino acid after the number the acquired residue. SPP = signal peptide peptidase; SP = signal peptidase; wt = Vp447; 8leu = Vp447_8leu_; Δc = Vp447_Δc_; Δ208–212 = Vp447_Δ208–212_.

**Figure 2:**
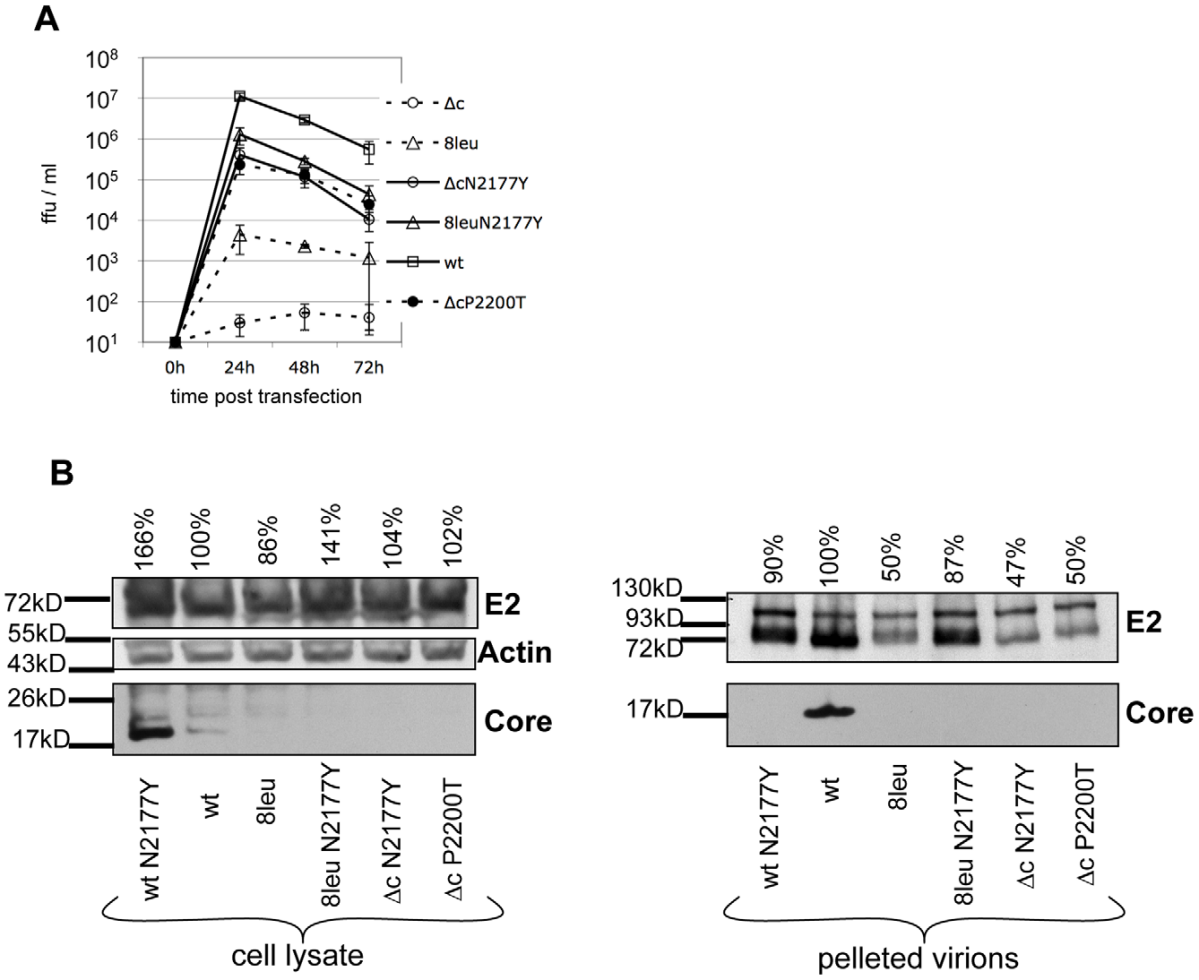
Growth of viruses encoding modifications of core or NS3 and detection of core protein and E2 glycoprotein in cell lysate and pelleted supernatant. (A) Virus titer (ffu/ml) was determined 24, 48 and 72 h after transfection of the respective viral genomes in SK6-cells. Depicted are mean and standard deviation of n = 3 experiments. (B) 72 h after transfection, SK6-cells and pelleted cell culture supernatant were lysed and subjected to Western Blot analysis. Amounts of E2 were quantified relative to Vp447 signal (set to 100%) and are indicated above the respective blots. wt = Vp447; wtN2177Y = Vp447N_2177_Y; 8leuN2177Y = Vp447_8leu_N_2177_Y; 8leu = Vp447_8leu_; ΔcN2177Y = Vp447_Δc_N_2177_Y; ΔcP2200T = Vp447_Δc_N_2200_T. Detection of β-actin served as loading control.

Recently, we reported that a single amino acid substitution (SAAS) (N_2256_Y) in the helicase domain of NS3 rescued a poorly growing CSFV construct (Vp447_Yc_) that encoded a core protein of which the N-terminus was fused to YFP [9]. This unexpected result prompted us to investigate spontaneously occurring revertants of a CSFV mutant in detail that initially was designed to determine requirements for core processing by signal peptide peptidase (SPP). Replacement of most of the signal peptide (aa 250–261) by a stretch of 8 leucine residues (Figure 1B) led to a poorly growing virus (4.5×10^3^ ffu/ml) (Vp447_8leu_) that showed a more than 200-fold rise in titer upon passaging in SK6 cells. To identify the genomic change(s) leading to virus rescue, virus progeny was repeatedly plaque-selected. Interestingly, sequence analysis of these selected viruses did not reveal changes in the genomic sequence of the mutated core. Rescue mutations were identified by reintroducing genomic fragments (nt31–1580; nt1480–3970; nt 3900–5570; nt 5500–8590; nt8330–10510; nt 10420–12290) of the rescued viruses into the parental plasmid p447_8leu_. Only introduction of a genomic fragment nt 5500–8590 encoding parts of NS3-NS4B (aa 1730–2656 of the polyprotein) into the parental plasmid resulted in rescue after transfection of the respective viral genomes. Upon sequencing of this fragment one SAAS was found in each clone tested in NS3 helicase subdomain 3 (namely E_2160_G, N_2177_Y, Q_2189_K, P_2200_T and N_2256_D) (Figure 1B). To prove that these SAAS were indeed responsible for the rescue, the respective mutations were each engineered into the full-length cDNA construct of Vp447_8leu_ (p447_8leu_E_2160_G, p447_8leu_N_2177_Y, p447_8leu_Q_2189_K, p447_8leu_P_2200_T, p447_8leu_N_2256_D). After transfection, the resulting viruses grew to titers exceeding 10^5^ ffu/ml without the need for passaging (Table 1). Growth characteristics are shown for the virus growing to highest titers (Vp447_8leu_N_2177_Y) (Figure 2A). The overall titer of Vp447_8leu_N_2177_Y was about one log_10_ below the one of Vp447. In the background of the parental Vp447, the N_2117_Y substitution led to a more than 20- fold decrease of virus output (Vp447N_2177_Y) in comparison to Vp447 (Table 1).

**Table 1:** Virus output of different viruses with modified core proteins depending on the amino acid exchange present in the NS3 helicase subdomain 3.

	wt	8leu	Δ208–212	Δc
-	1.1×10^7^	4.5×10^3^	7.1×10^2^	3×10^1^
E_2160_G		2.2×10^5^	ND	4.5×10^4^
N_2177_H		ND	7.9×10^5^	2.8×10^4^
N_2177_Y	3.3×10^5^	1.3×10^6^	ND	4.0×10^5^
Q_2189_K		3.4×10^5^	ND	1.3×10^4^
P_2200_T		8.0×10^5^	ND	2.3×10^5^
N_2256_D		4.9×10^5^	ND	7.1×10^4^

Virus content in the supernatant in ffu/ml 24 h after transfection of the respective viral genomes in SK6-cells. wt = Vp447; 8leu = Vp447_8leu_; Δc = Vp447_Δc_; Δ208–212 = Vp447_Δ208–212_; ND = not done.

To assess whether acquisition of SAAS in NS3 helicase subdomain 3 might be a general mechanism of CSFV to overcome defects in the core gene, rescue experiments with a different loss of core function mutant were attempted. An initially poorly growing CSFV (7.1×10^2^ ffu/ml 24 h after transfection) encoding an internal deletion (aa 208–212) in the core gene (Vp447_Δ208–212_) (Figure 1C) was passaged in SK6 cells until an increase in virus growth was observed. Using the same approach as described above, a SAAS at position N_2177_H was identified. After introducing this SAAS N_2177_H into parental plasmid, virus titer (Vp447_Δ208–212_N_2177_H) rose to 7.9×10^5^ ffu/ml 24 h after transfection of the respective virus genome in SK6 cells (Table 1). Apparently single amino acid substitutions in the C-terminal subdomain of the NS3 helicase compensate for functionally compromised core mutants that are compromised by N-terminal fusion to YFP (Vp447_Yc_), defective C-terminal processing (Vp447_8leu_) or an internal deletion (Vp447_Δ208–212_), respectively.

Core protein can be detected in lysates of cells transfected with genome of Vp447 and in pelleted virions of Vp447 (Figure 2B). Surprisingly, Western blot analysis of cell lysate and pelleted virus particles revealed that neither Vp447N_2177_Y nor Vp447_8Leu_N_2177_Y contained detectable levels of core protein in concentrated virus preparations. Core protein could be detected in lysates of SK6 cells transfected with genome of Vp447N_2177_Y, but not after transfection of genomes of Vp447_8leu_ and Vp447_8leu_N_2177_Y.

### Rescue of a core deletion mutant (Vp447_Δc_)

Mutations within the NS3 helicase subdomain 3 allowed the rescue of viruses with compromised core function. To examine whether the core-coding region is dispensable altogether, almost the entire core gene (aa170–246; 77 of the 86 codons) was deleted in p447, yielding p447_Δc_ (Figure 1D). Nine C-terminal amino acids (247–255: LEKALLAWA) were preserved as part of the signal sequence (aa 247–269) to ensure translocation of E^rns^ into the ER lumen. While this construct lacking the core-coding region was not viable, introduction of above described SAAS in NS3 into p447_Δc_ (p447_Δc_E_2160_G, p447_Δc_N_2177_H, p447_Δc_N_2177_Y, p447_Δc_Q_2189_K, p447_Δc_P_2200_T, p447_Δc_N_2256_D) led to the release of infectious virus with titers of at least 1×10^4^ ffu/ml 24 h after electroporation of the respective transcripts (Table 1). Highest titers were observed for Vp447_Δc_N_2177_Y and Vp447_Δc_P_2200_T (4.0×10^5^ and 2.3×10^5^ ffu/ml 24 h after transfection in SK6 cells), thus being 30–50 -fold below Vp447 titer (Figure 2A). Hence, SAAS in the helicase domain of NS3 can not only compensate for functionally compromised, but even completely absent core protein. No upstream open reading frame longer than 15 codons that might provide the virus with an alternative core protein could be identified. As expected, no core protein could be detected in either cell lysate or supernatant of RNA cells transfected with Vp447_Δc_N_2177_Y or Vp447_Δc_P_2200_T (Figure 2B). To exclude a possible function of the C-terminal core aa 247–269 in Vp447_Δc_N_2177_Y, they were replaced by the signal peptide of bovine CD46, a cell surface glycoprotein (Vp447_Δc_N_2177_Y_CD46SP_). Progeny virus production of Vp447_Δc_N_2177_Y_CD46SP_ was slightly reduced (1×10^5^ ffu/ml 24 h after transfection) in comparison to Vp447_Δc_N_2177_Y. Analysis of cell lysate of Vp447_Δc_N_2177_Y 72 h after transfection of SK6 cells did not reveal differences in the relative presence and processing of NS2–3, NS5B, E^rns^ and E2 in comparison to wildtype (Figure S1). This suggests that cellular protein expression and polyprotein processing is neither affected by the lack of core protein nor by the presence of a SAAS in the NS3 helicase. The relative reduction of protein expression in Vp447_Δc_ genome transfected cells results from its inability to spread. No changes in the regions surrounding the deletion of the core gene and NS3 were detected after ten passages of Vp447_Δc_N_2177_Y in SK6 cells (data not shown). Introduction of combinations of the described amino acid exchanges in NS3 helicase of Vp447_Δc_ showed no additive effect but rather resulted in a 10–100 fold drop in virus titer (data not shown).

### Phenotypic characterization of Vp447_Δc_N_2177_Y

**Figure 3:**
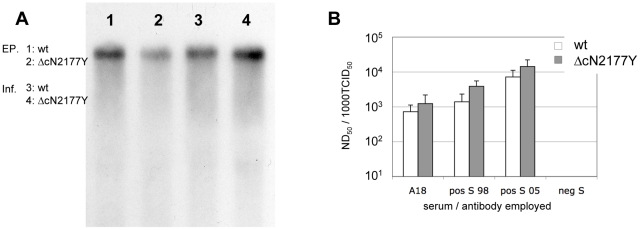
Genome detection and neutralization of Vp447_Δc_N_2177_Y. (A) Whole cellular RNA of SK6-cells either transfected with the genome of Vp447 or Vp447_Δc_N_2177_Y or infected with Vp447 or Vp447_Δc_N_2177_Y was subjected to Northern blot analysis. The size of the viral genomic RNA is indicated above the arrows. (B) Reduction of infectivity in ND_50_/1000 TCID_50_ upon incubation with Vp447 or Vp447_Δc_N_2177_Y with a monoclonal antibody against E2 or sera of vaccinated/infected animals. EP = RNA of cells transfected with viral genomes; Inf = RNA of cells infected with either Vp447 or Vp447_Δc_N_2177_Y; wt = Vp447; ΔcN2177Y = Vp447_Δc_N_2177_Y.

The lack of a structural component of the virus particle may result in altered phenotypic properties of the virus. We therefore assessed virus infectivity, morphology, and physical stability of Vp447_Δc_N_2177_Y compared to wildtype Vp447. The presence of viral genome in cells transfected with genomic RNA of Vp447 or Vp447_Δc_N_2177_Y or infected with Vp447 or Vp447_Δc_N_2177_Y was assessed by Northern blot analysis. Genomes could be detected for Vp447_Δc_N_2177_Y (12059 nt) and Vp447 (12293 nt) (Figure 3A), but the size difference of 234 nt could not be resolved. To verify that the infectivity of Vp447_Δc_N_2177_Y is due to proper virus particles, not secreted replication complexes, neutralization assays were performed. Incubation of Vp447_Δc_N_2177_Y with either a monoclonal antibody against E2 (A18) or sera of one vaccinated animal (S98) and one vaccinated and subsequently CSFV infected animal (S05) neutralized infectivity in the same fashion as observed for the parental Vp447 (Figure 3B). Next, specific infectivity in the supernatant was assessed. To allow for strict discrimination between both viruses on the level of RNA, a modified Vp447_Δc_N_2177_Y, encoding for 5 alanine residues between the N^pro^ C-terminus and core residue 247 (Vp447_Δc+5Ala_N_2177_Y) was generated. The resulting PCR assay specifically amplified either Vp447 or Vp447_Δc+5Ala_N_2177_Y genomes (Figure S2). In cell culture, growth of Vp447_Δc+5Ala_N_2177_Y was slightly improved in comparison to Vp447_Δc_N_2177_Y. With this approach, we determined a specific infectivity (ratio of virus genomes versus infectivity in cell culture supernatant) of 23 genomes/ffu (SD±14; n = 3) for Vp447 and 131 genomes/ffu (SD±61; n = 3) for Vp447_Δc+5Ala_N_2177_Y.

**Figure 4:**
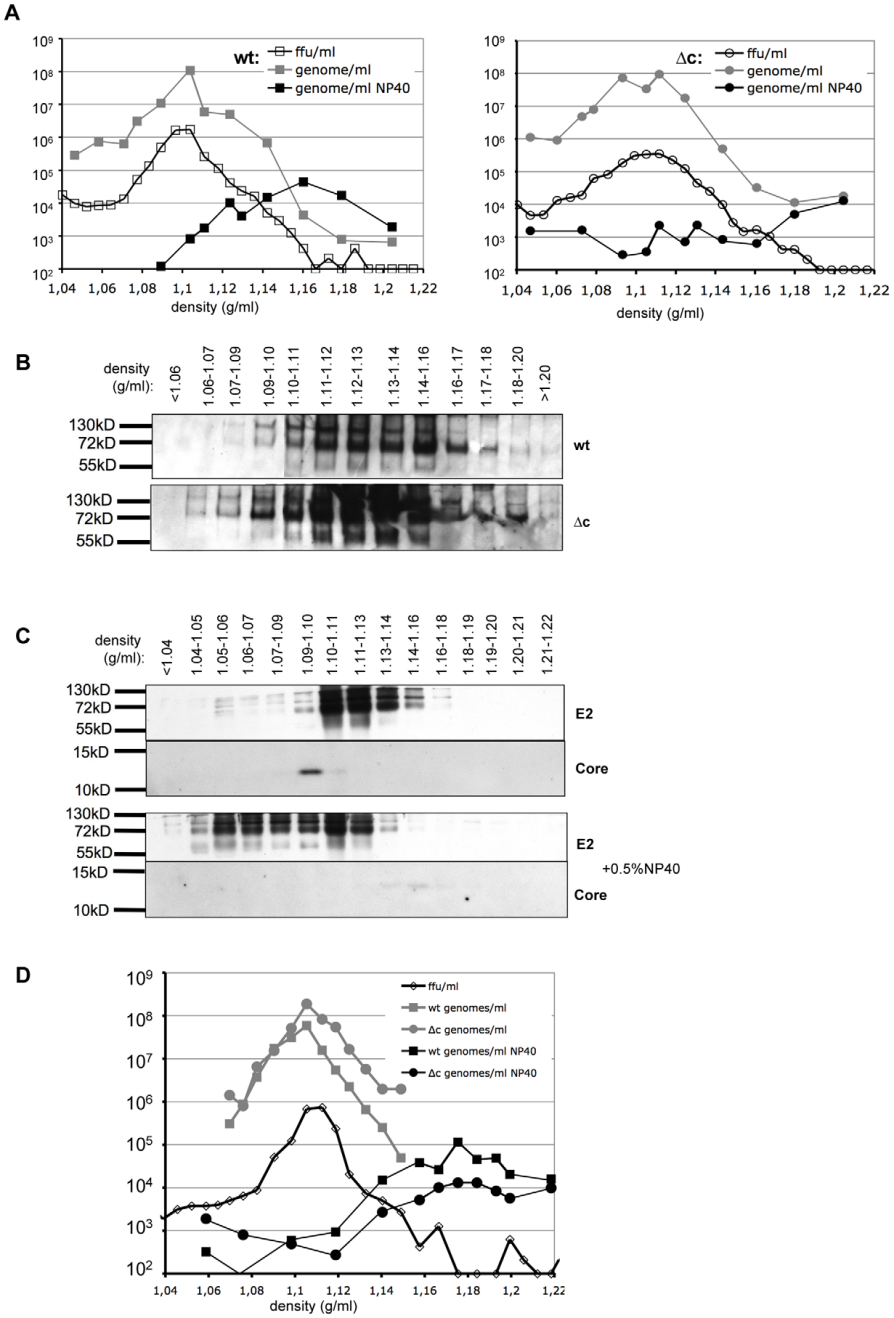
Determination of density and behaviour upon detergent treatment of Vp447 and Vp447_Δc_N_2177_Y. (A) Viral RNA content and infectivity was determined according to density for Vp447 (wt) and Vp447_Δc_N_2177_Y (Δc) on separate gradients with or without previous treatment with 0.5% NP40. (B) Additionally, the distribution of E2 was analyzed according to density in Western Blot for both viruses. Subsequently, both viruses were separated on the same gradient with and without treatment with 0.5% NP40. (C) Subsequently, the distribution of E2 and core was determined according to density, (D) as was infectivity and RNA content.

To determine density and size of Vp447 in comparison to Vp447_Δc_N_2177_Y, equilibrium density centrifugation and size exclusion chromatography was performed. The densities of Vp447 and Vp447_Δc+5Ala_N_2177_Y were compared by separation in individual, continuous sucrose gradients (10–60%) and equilibrium centrifugation. 30 fractions of 360 µl each were harvested by bottom puncture. In repetitive experiments, infectivity peaked at a density of 1.104–1.111 g/ml for Vp447 and of 1.099–1.112 g/ml for Vp447_Δc+5Ala_N_2177_Y (Figure 4A). RNA levels, determined by virus specific real-time RT-PCR, peaked at a density of 1.10 g/ml for Vp447 and at 1.09–1.11 for Vp447_Δc+5Ala_N_2177_Y (Figure 4A). Peak E2 levels were detected from 1.10–1.14 g/ml for both viruses, but E2 was present in all fractions (Figure 4B). To avoid variations between two gradients, 10^6^ ffu of both viruses were mixed and layered on top of the same sucrose gradient. As described above, 30 fractions of 360 µl each were harvested by bottom puncture. Again, E2 was detectable over a wide range of the gradient (1.04–1.18 g/ml sucrose) (Figure 4C) and infectivity peaked at a density of 1.105–1.113 g/ml (Figure 4D). Highest levels of Core protein were detectable at a density of 1.09–1.10 g/ml. RNA levels of either virus matched with infectivity and peaked in the same fraction (1.105 g/ml) (Figure 4D).

To address the effect of the SAAS N_2177_Y in Vp447_Δc_ on particle formation, Vp447_Δc+5Ala_N_2177_ was created. 75 ml of supernatant of SK6-cells 48 h after transfection with genomes of either Vp447_Δc+5Ala_N_2177_ or Vp447_Δc+5Ala_N_2177_Y were subjected to equilibrium centrifugation (Figure S3). Highest levels of infectivity were recorded at a density of 1.117 g/ml for Vp447_Δc+5Ala_N_2177_Y and at 1.102 g/ml for Vp447_Δc+5Ala_N_2177_. Both infectivity and RNA-levels were reduced more than 400-fold in Vp447_Δc+5Ala_N_2177_ in comparison to Vp447_Δc+5Ala_N_2177_Y in all fractions tested. Overall, E2 levels were comparable between both viruses and peaked at 1.12–1.14 g/ml. However, the ratio of E2 homo- to heterodimer seemed to differ between the two viruses, as did the E2 levels at a density of 1.10 g/ml.

The nucleocapsid of Vp447 is likely composed of core protein and the viral genome but so far has not been characterized. To gain at least preliminary information about the nucleocapsid of Vp447 and whether an analogous structure exists in Vp447_Δc+5Ala_N_2177_Y, either virus was treated with a nonionic detergent (0.5% NP40) to remove the envelope prior to equilibrium centrifugation as described above. The treatment completely abrogated infectivity in the fractions recovered and viral RNA levels were reduced more than 100-fold for either virus in comparison to untreated virus. RNA levels were just above background and peak levels occurred at densities of 1.05 g/ml and 1.2 g/ml for Vp447_Δc+5Ala_N_2177_Y whereas a broad peak of genomic RNA could be detected at densities of 1.11–1.2 g/ml for Vp447 (Figure 4A). To increase precision of the analysis, both viruses were mixed, treated with 0.5% NP40 and analyzed in the same gradient. The E2 signal was shifted towards the top of the gradient (1.04–1.14 g/ml), whereas weak core signals could be detected at higher densities (1.13–1.18 g/ml) (Figure 4C). Viral genome of Vp447 was detected in highest amounts at densities of 1.14–1.2 g/ml, whereas highest levels of Vp447_Δc+5Ala_N_2177_Y genome were now observed at densities of 1.17–1.19 g/ml and 1.22 g/ml (Figure 4D). These results indicate that detergent treatment of Vp447 in fact releases nucleocapsids of higher density. This assay is complicated by the RNase activity of the structural protein E^rns^, which might result in degradation of the viral genome after lysis of the lipid envelope. Hence, both Vp447 (Vp447_H30K) and Vp447_Δc+5Ala_N_2177_Y (Vp447_Δc+5Ala_N_2177_Y_H30K) with an exchange of E^rns^ residue histidine 30 to arginine, destroying the active centre of its RNase, were generated [25]. This aa exchange did not affect the amount of progeny virus produced (Figure S4, Figure S5). Both viruses were subjected to equilibrium density centrifugation to compare them with the respective parental virus. No differences were present regarding the amount and distribution of E2 (Figure S4; data for Vp447_Δc+5Ala_N_2177_Y_H30K not shown). After detergent treatment, RNA levels of Vp447 and Vp447_H30K as well as of Vp447_Δc+5Ala_N_2177_Y and Vp447_Δc+5Ala_N_2177_Y_H30K remained at low levels (Figure S4, Figure S5).

**Figure 5:**
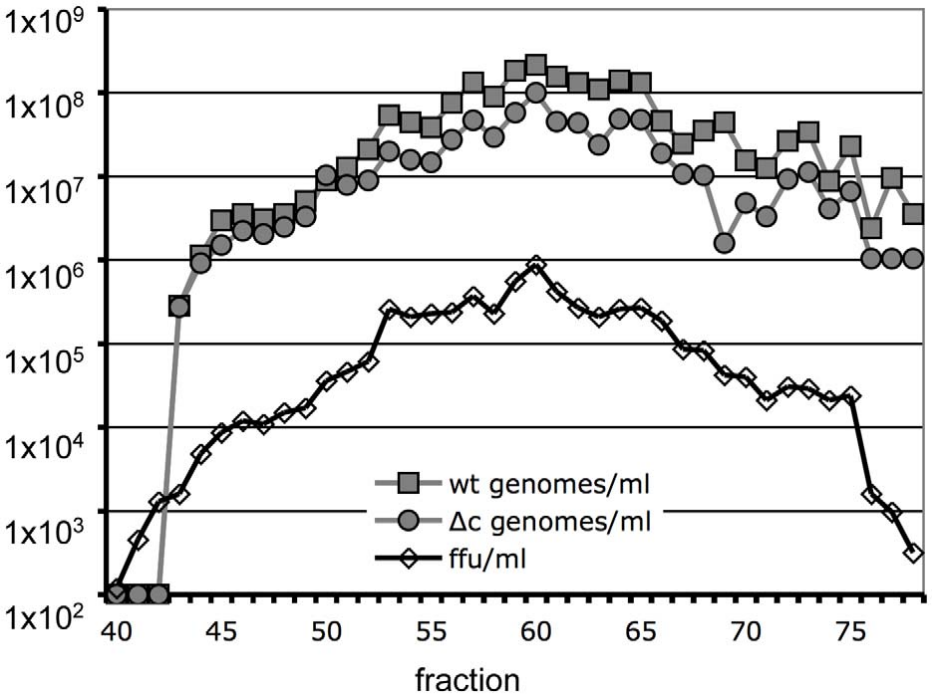
Distribution of infectivity and genomes of Vp447 and Vp447_Δc_N_2177_Y upon size exclusion chromatography. 10^8^ ffu/ml of Vp447 and Vp447_Δc_N_2177_Y each were simultaneously applied to a size exclusion chromatography column and virus titer was determined for all fractions. Genomes of both viruses were quantified in the different fractions by virus specific real-time RT-PCRs. The column was calibrated employing IgM as a size marker. wt = Vp447; Δc = Vp447_Δc_N_2177_Y.

Size exclusion chromatography was performed to directly compare the Stokes diameter of Vp447 and Vp447_Δc+5Ala_N_2177_Y. For this purpose, a mixture of 10^8^ ffu of each Vp447 and Vp447_Δc+5Ala_N_2177_Y was subjected to gel filtration using Superose 6. Infectivity was detectable in fractions 40–78. Real-time RT-PCR (as described above) differentiating Vp447 from Vp447_Δc+5Ala_N_2177_Y allowed detection of viral genomes in fractions 43–78. Peak levels of genomes of either virus were observed in fractions 59–61 and coincided with peak infectivity (Figure 5).

**Figure 6:**
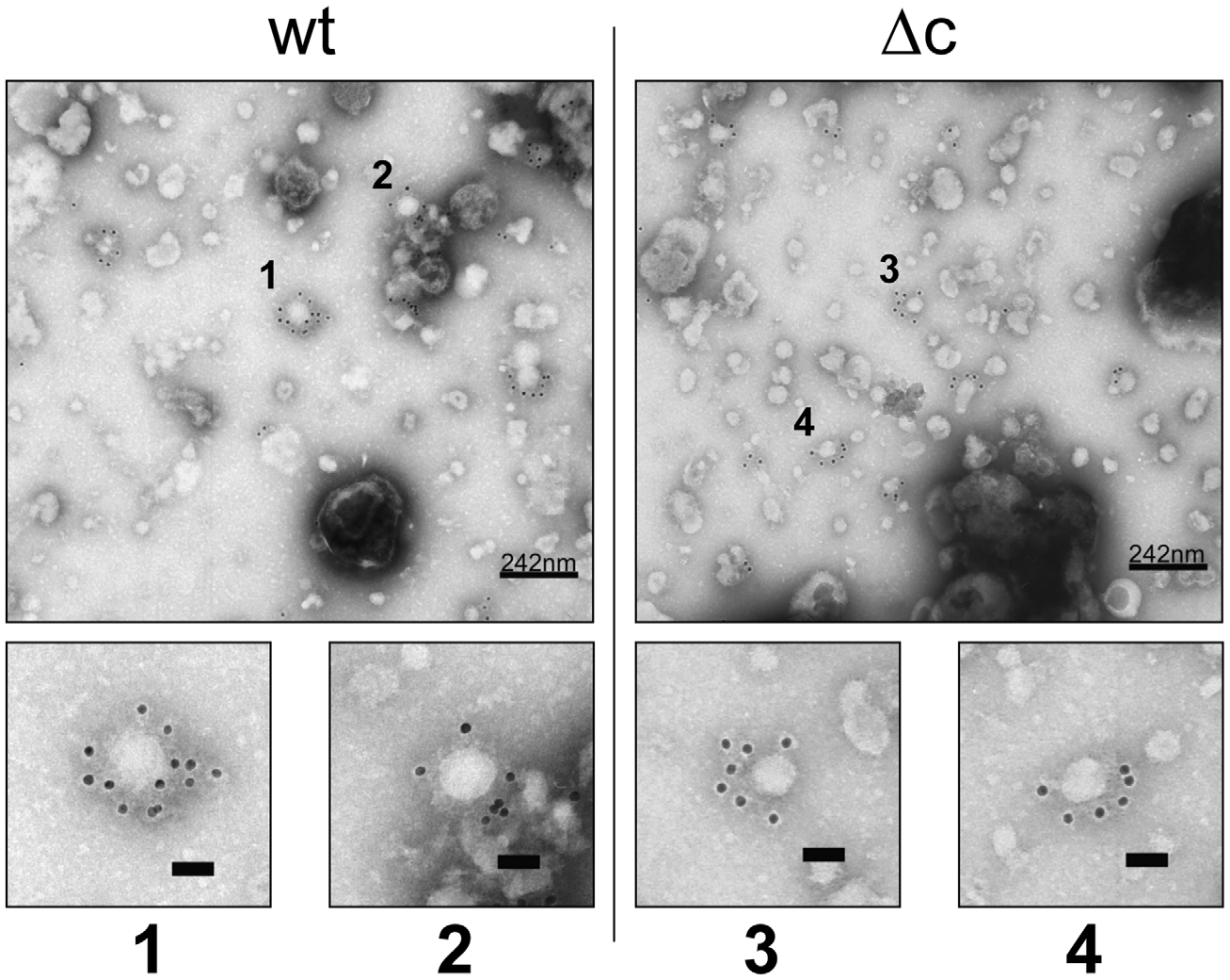
Morphology of Vp447 (wt) and Vp447_Δc_N_2177_Y (Δc) particles. Serum free virus preparations were subjected to negative stain electron microscopy after concentration by ultracentrifugation. To confirm the observed virus like particles, they were immunogold labeled against the viral glycoprotein E^rns^. Numbers indicate particles magnified from the original image. Bar in the magnified images represents 50 nm.

For electron microscopic inspection, virus was produced in SK6 cells in serum free medium, concentrated by ultracentrifugation and inspected by TEM. The identity of the virions was confirmed by immunogold (10 nm) staining with a monospecific rabbit serum against E^rns^ (for specificity of this serum, see Figure S6). In both preparations, pleomorphic particles of about 50 nm were detectable. No morphological changes were apparent between Vp447 and Vp447_Δc_N_2177_Y particles (Figure 6). Mean size of Vp447 particles was 51.9 nm (standard deviation 8.9 nm; n = 43) and of Vp1017 particles 50.1 nm (standard deviation 9.3 nm; n = 34). However, no exact size comparison or tomographic particle analysis was possible since required particle quantity, quality and purity was not achieved.

To address whether the absence of core protein in the virus particle affects physical stability of Vp447_Δc_N_2177_Y, the kinetics of inactivation of Vp447 and Vp447_Δc_N_2177_Y at 37°C and 39.5°C were determined. No major differences in thermal stability were observed between the two viruses (Figure S7). Physical stability was also assessed by freezing and thawing of defined virus preparations. After thawing, 19% of the initial virus input could be recovered for Vp447 and 13% for Vp447_Δc_N_2177_Y (Figure S7).

### Vp447_Δc_N_2177_Y is avirulent

**Figure 7:**
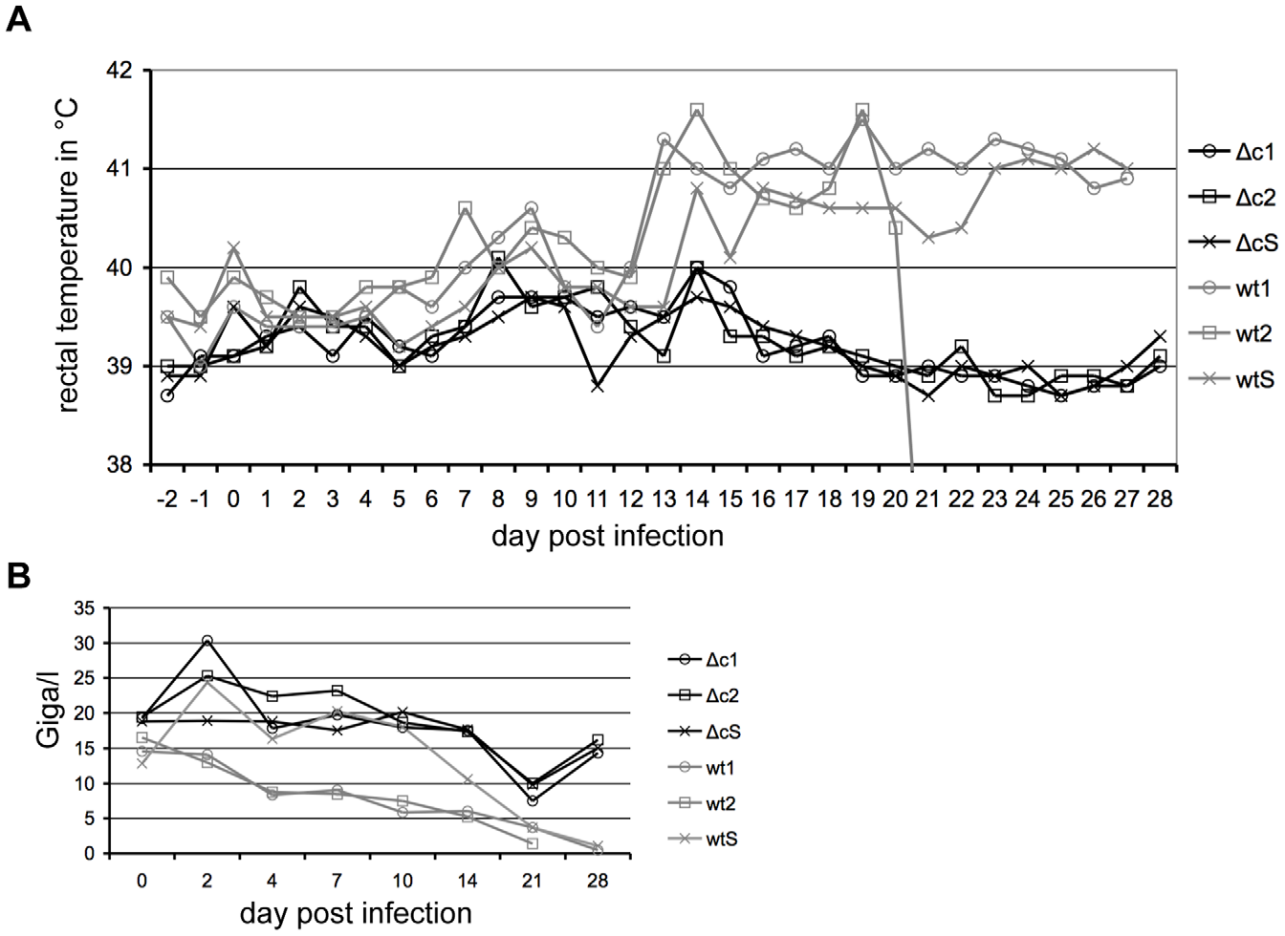
Temperature and blood leukocyte counts of pigs infected with 5×10^6^ TCID_50_ Vp447 or Vp447_Δc_N_2177_Y. After infection, rectal temperature (A) was recorded daily and EDTA blood was collected on days 2, 4, 7, 10, 14, 21 and 28 and the number of leukocytes (B) was determined in Giga/l. wt = Vp447 infected; Δc = Vp447_Δc_N_2177_Y infected; wtS = sentinel animal in Vp447 infected group; ΔcS = sentinel in Vp447_Δc_N_2177_Y infected group.

CSF is a disease of pigs with strain dependent virulence. Vp447 represents a moderately virulent strain [26], causing mortality rates >50%. To assess virulence of Vp447_Δc_N_2177_Y, a small-scale animal experiment was conducted. Two groups of two pigs each were injected intramuscularly with 5×10^6^ TCID_50_ of Vp447 or Vp447_Δc_N_2177_Y. Two days later, a sentinel pig was added to each group. Animals were evaluated according to a standard clinical scoring system [27], rectal temperature and leukocyte counts. Vp447 infected animals exhibited febrile temperatures (>40°C) on day 7–10 after infection and from day 13 after infection until the end of the experiment (Figure 7A). One Vp447 infected pig (wt2) had to be euthanized on day 21 after infection, with a clinical score of 10. The other Vp447 infected pig (wt1) had a clinical score between 2.5 and 4.5 on days 17, 18 and 21–27. Severe leukopenia (leukocyte count below 10 Giga/l), a typical symptom of CSF [reviewed with other clinical symptoms by 28], was present in wt1 and wt2 from day 4 after infection, with further declining leukocyte counts until the end of the experiment (Figure 7B). The sentinel animal (wtS) housed together with the Vp447 infected pigs developed febrile temperatures from day 14 after infection until the end of the experiment and leukopenia was present on day 21 and 28 of the experiment. Virus could be isolated from Vp447 infected animals on days 4, 7, 10 and 14 after infection (Table 2). Virus isolation was not possible from the sentinel animal on days 4, 7, 10 and 14 after infection of the other pigs. Neutralizing antibodies could not be detected in Vp447 infected animals and their sentinel on days 10, 14 and 21 after infection (Table 3). No apparent signs of disease (clinical score = 0) were observed for Vp447_Δc_N_2177_Y infected animals (Δc1 and Δc2) and their sentinel (ΔcS) throughout the experiment. With the exception of one day of slightly elevated body temperature (Δc2 on day 8) and mild leukopenia of animal Δc1 on day 21, no fever or leukopenia were present in Vp447_Δc_N_2177_Y infected animals (Δc1 and Δc2) and their sentinel (ΔcS). We were unable to reisolate Vp447_Δc_N_2177_Y from sera (Table 2) and leukocytes (not shown) of infected animals on days 2, 4, 7, 10 and 14 after infection. However, viral genomes could be amplified from leukocytes until day 7 and neutralizing antibodies could be detected beginning with day 14 after infection (Table 3).

**Table 2:** Recovery of virus from sera on SK6/Rie 5-1 cells at day (d) 2, 4, 7, 10 and 14 after infection.

	Δc1	Δc2	ΔcS	wt1	wt2	wtS
**d2**	neg/neg	neg/neg	neg/neg	neg/neg	neg/neg	neg/NR
**d4**	neg/neg	neg/neg	neg/neg	pos/pos	neg/NR	neg/neg
**d7**	neg/neg	neg/neg	neg/NR	pos/pos	pos/pos	neg/neg
**d10**	neg/neg	neg/NR	neg/NR	pos/NR	pos/NR	neg/neg
**d14**	neg/neg	neg/neg	neg/neg	pos/pos	pos/pos	neg/neg

wt = Vp447 infected; Δc = Vp447_Δc_N_2177_Y infected; wtS = sentinel animal in Vp447 infected group; ΔcS = sentinel in Vp447_Δc_N_2177_Y infected group. Neg = no virus isolated; pos = virus isolated; NR = not readable.

**Table 3:** Titer of neutralizing antibodies in swine sera on day (d) 0, 10, 14 and 21 after infection in ND_50_/ml.

	Δc1	Δc2	ΔcS	wt1	wt2	wtS
**d0**	<5	<5	<5	<5	<5	<5
**d10**	<5	<5	<5	<5	<5	<5
**d14**	7.5	<5	<5	<5	<5	<5
**d21**	316.23	158.49	<5	<5	<5	<5

wt = Vp447 infected; Δc = Vp447_Δc_N_2177_Y infected; wtS = sentinel animal in Vp447 infected group; ΔcS = sentinel in Vp447_Δc_N_2177_Y infected group.

## Discussion

Key findings of this study are that (1) a pestivirus lacking almost the entire core coding region is viable and that (2) viability depends on single point mutations in the helicase domain of NS3. This finding questions the general assumption that a core protein is a specific and essential structural element of enveloped RNA viruses and is supported by the existence of GBV- A and GBV- C, which do not encode an obvious core protein [reviewed by 1]. Further to this, the data support a central role of the multifunctional NS3 protein in virus particle assembly.

During the characterization of different loss - of - function manipulations of the core gene of CSFV, we observed that some replicative but initially poorly growing viruses generated increased amounts of progeny virus after extended incubation periods of the transfected cells. The responsible gain-of-function mutations could not be mapped to the locus of the manipulated nucleotide sequence. Instead, single nucleotide exchanges clustered within a stretch of approximately 300 nucleotides of NS3 helicase subdomain 3, about 6000 nucleotides downstream of the core gene. The occurrence of second site mutations in NS3 upon loss of core protein function differs from results described for tick-borne encephalitis virus. In this model, the deletion of parts of the internal hydrophobic domain led to the acquisition of hydrophobic residues in the core gene itself [29].

To confirm that the observed infectivity of core deficient viruses was due to proper virus particles, Vp447 and Vp447_Δc_N_2177_Y were compared with regard to sensitivity towards neutralizing antibodies. In both cases, infectivity was blocked by hyperimmune sera from pigs or a monoclonal antibody directed against viral E2. Differences in the stability of particles of Vp447 and Vp447_Δc_N_2177_Y with regard to infectivity were not observed upon freezing - thawing and heat exposure. Electron micrographs of Vp447 and Vp447_Δc_N_2177_Y were obtained from concentrated serum-free cell culture supernatants and the structures observed were immunogold labelled with a monospecific rabbit serum against E^rns^. This was necessary because pestivirions in general lack a characteristic morphology. No morphological differences between Vp447 and Vp447_Δc_N_2177_Y particles were apparent. Precise determination of structure and size would require cryo EM to avoid preparation dependent artifacts and also larger numbers of particles.

With regard to particle sizes no apparent differences in Stokes diameter could be detected between Vp447 and Vp447_Δc_N_2177_Y particles in gel filtration experiments. Both viruses eluted from the column in the same fractions. Due to difficulties in comparing different gel filtration runs, it was mandatory to separate Vp447 and Vp447_Δc_N_2177_Y side by side. To distinguish between both viruses by real time RT-PCR, a modified Vp447_Δc_N_2177_Y was constructed, which encodes an additional sequence of five alanines between N^pro^ C-terminus and signal peptide (Vp447_Δc+5Ala_N_2177_Y). This construct was also employed for determination of virus density in linear sucrose gradients. After it was evident from individual gradient experiments that infectivity and genomic RNA comigrated at densities from 1.10–1.11 g/ml (Figure 4), both viruses were mixed and analyzed in the same gradient. Again RNA, E2 and infectivity accumulated at the same densities. A surprising finding was that core protein showed highest concentration at slightly lower densities than peak RNA and infectivity levels. As E2 can be detected in the supernatant of cells transfected with the genome of Vp447_Δc_, it was of interest to compare the suspected pseudoparticles with regard to density and genome integration to Vp447_Δc_N_2177_Y. Hence, equal volumes of supernatant of cells either transfected with Vp447_Δc+5Ala_N_2177_ or Vp447_Δc+5Ala_N_2177_Y genomes were subjected to density gradient centrifugation. The reduction of infectivity of Vp447_Δc+5Ala_N_2177_ in comparison to Vp447_Δc+5Ala_N_2177_Y correlated with the reduction of genome levels at the densities tested, suggesting that the SAAS N_2177_Y is critical for the integration of the virus genome into the particles during assembly if core protein is not present. Overall, E2 levels between the two viruses were comparable both with regard to total amount in the supernatant and distribution according to density. However, the ratio of E2 homo- to heterodimer, as well as the amounts of E2 at a density of 1.1 g/ml and the density of peak infectivity differed between the two viruses, which might indicate differences in particle composition.

To determine whether core protein actually is a component of a nucleocapsid structure, the envelope of the virus particles was removed by treatment with a non-ionic detergent (Nonidet P40). NP40 treated viruses were layered on top of sucrose gradients as before and the position of infectivity, E2, core and RNA were recorded after equilibrium centrifugation. Infectivity could be abolished completely by NP40 treatment. The signal of E2 shifted towards the top of the gradient (1.04–1.14) whereas the core protein signal shifted to higher densities (1.13–1.18). Peak values of viral genomes coincided with core signal in Vp447, which might implicate the presence of a nucleocapsid like structure of higher density. For Vp447_Δc+5Ala_N_2177_Y, signals for viral genome were low, with a slight elevation at the tube bottom if the virus was separately run on a gradient. Hence, we were unable to assign the genome to a discrete density. In contrast, a slight peak of viral RNA, comparable in density to Vp447, was observed if both viruses were separated in the same gradient. One could speculate that this effect is due to a redistribution of core protein between viral genomes after detergent treatment. Overall, the amounts of RNA determined by real time RT-PCR were 10^2^–10^4^ lower than with intact viruses, which can be taken as evidence for RNA degradation. A comparable experiment for HCV determined only a six-fold reduction of genomic RNA after NP40 treatment [30]. A major difference between HCV and CSFV is the presence of the potent ribonuclease E^rns^ in the virus envelope [31]. However, after mutational disruption of the RNase active centre of E^rns^ [25], we did not observe changes in the levels of viral genome detectable in comparison to virus with intact RNase. This suggests that the analytic system itself, by employing sucrose, contains RNases, which together with the long centrifugation time (24 h), are sufficient to degrade most of the viral genomes present in the sample. To address this technical problem, improved separation methods have to be established to minimize RNA degradation. However, the relatively higher amount of viral genome detectable for Vp447 in comparison to Vp447_Δc_N_2177_Y suggests a protective function of core protein against RNase.

The absence of core protein and thus a known proteinaceous component of the nucleocapsid questions the way how a linear viral RNA molecule of approximately 3 µm is condensed in order to fit into the virus particle of less than 50 nm diameter. Further to this, the genome has a negative charge that is partially neutralized by a usually positively charged (nucleo-) protein. Strikingly, introduction of the single amino acid substitution N_2177_Y into the parental Vp447 (Vp447N_2177_Y) reduced virus growth and abrogated the detectable incorporation of core protein into the virus particles, while at the same time the core protein accumulated intracellularly. This points to an ability of modified NS3 to counteract core particle integration, probably by modulation of core-RNA-interaction. This finding also raises the question whether NS3 might replace core in the virus particle. So far, we were unable to detect any NS3 in purified virus preparations, but we cannot exclude that a small number of molecules is packaged.

As we have no evidence for other virally encoded proteins for replacement of the missing core protein, it is conceivable that host cellular proteins, for example cytoplasmic RNA chaperones or nuclear RNA binding proteins, compensate for the lack of core protein. The association of cellular proteins with virus particles has been described for RNA and DNA viruses, like hepadnaviruses [32], rabies virus [33], filoviruses [34], respiratory syncytial virus [35] and HCV [36]. Interestingly, HSP70 or HSP90 were most often found associated with virus particles. An important task will therefore be a proteome analysis of highly purified virus particles of Vp447 and Vp447_Δc_N_2177_Y. Epitope tagged viruses - as described for HCV [37], [38] and BVDV [39] - may be useful for such an investigation.

NS3 is functionally well conserved among members of the *Flaviviridae* and significant sequence conservation is apparent. It is a multifunctional protein that contains several enzymatic activities, such as serine protease, NTPase and RNA helicase [18]–[22]. Its involvement in particle assembly has been suggested for HCV [11], [13] and YFV [12], [40], [41]. The conserved helicase motifs are located in subdomains 1 and 2 of the NS3 helicase [42]. NS3 helicase subdomain 3 is the least conserved stretch in NS3 of *Flaviviridae*, both with regard to amino acid sequence and structure [43]. Although it is not present in all superfamily 2 helicases [44], it is essential for NS3 helicase activity. Analysis of all single aa substitutions in the putative CSFV NS3 helicase subdomain 3, which were able to rescue Vp447_Δc_N_2177_Y, did not reveal an obvious pattern with regard to amino acid identity, charge or polarity, hence we are not able to draw conclusions about the mode of action by analysis of the sequence identities. So far, the 3D-structure of pestiviral NS3 helicase is not known and the sequence homology to HCV NS3 is too low to draw conclusions. All rescue mutations were located in regions aligning with alpha helices both in dengue virus [45] and HCV [46], [47] (Figure S8). All but one aa substitution identified were located in stretches reported to be important for NS3 helicase protein-protein-interaction and optimal replication of HCV [48]. So far, there is no mechanistic explanation how the described mutations in NS3 helicase domain 3 allow for the rescue of Vp447_Δc_. Structural and functional analysis of the modified NS3 proteins are needed to elucidate the gain of function in particle assembly.

Finally, the virulence of Vp447_Δc_N_2177_Y in comparison to Vp447 was assessed in a small scale animal experiment. The parental CSFV strain used for this study causes disease in pigs with a case fatality rate of >50% [26]. While the two pigs infected with Vp447 and the sentinel housed together with these two pigs developed typical signs of CSF, the pigs infected with Vp447_Δc_N_2177_Y and the respective sentinel animal stayed completely healthy although they were injected with the same dose of virus. Neither fever nor leukopenia was observed in pigs infected with Vp447_Δc_N_2177_Y. Detection of genomic RNA in leukocytes up to day 7 p.i. and the appearance of CSFV neutralizing antibodies in both Vp447_Δc_N_2177_Y infected animals beginning at day 14 suggest that a limited replication took place in the animals, despite our inability to reisolate Vp447_Δc_N_2177_Y from serum or blood cells. This indicates that the lack of core protein leads to a strong attenuation of the virus. The sentinel pig developed no neutralizing antibodies, which can be taken as evidence that Vp447_Δc_N_2177_Y is not or inefficiently transmitted. All this points to an important role of pestiviral core protein in vivo. Further effort will be put in the characterization of Vp447_Δc_N_2177_Y in primary cells of its natural host to elucidate the mechanisms underlying its attenuation.

## Materials and Methods

### Ethics statement

All animal work was conducted according to the legal regulations of the German Animal Welfare jurisdiction (Tierschutzgesetz). The animal experiment was subject to authorization and was recorded after approval under reference number AZ 06/1105 at the Lower Saxony State Office for consumer protection and food safety. The internal reference was V2006-6.

### Generation of recombinant CSFVs

Sequence modifications were introduced into the core or NS3 protein of CSFV Alfort/Tübingen recombinant full length cDNA clone (p447) by site directed mutagenesis or end to end ligation, utilizing *Pfu*-DNA polymerase (Promega, Mannheim, Germany) (Primers are available upon request). Sequence analysis was employed to confirm the generated constructs (Quiagen, Hilden, Germany).

### Cell culture and virus rescue

SK6-cells were grown in Dulbecco's modified Eagle's medium supplemented with 10% fetal calf serum at 37°C under 5% CO_2_. Virus cDNA was transcribed into RNA using *SP6*-polymerase (NEB, Frankfurt am Main, Germany) and, typically, 2.5 µg RNA were electroporated into 5×10^6^ SK6-cells (Bio-Rad Gene Pulser). Replication was assessed 14 h after electroporation via immunohistochemistry using monoclonal antibody A18, directed against the CSFV E2 protein. Virus titer was determined in focus-forming units/ml (ffu/ml) 24 h after electroporation. For this purpose, supernatant was harvested, clarified (5 min at 3,000×g), and seeded on SK6-cells, employing 10-fold dilution steps. After 14 h, cells were fixed and stained for E2 as mentioned above. Antigen-positive foci of infected cells were counted using a Nikon Eclipse TS100 microscope and the titer was calculated. All virus titers were confirmed by multiple experiments (more than two).

For virus passaging, cell culture supernatant was harvested 72 h after electroporation of genomic RNA and clarified by centrifugation (5 min at 3,000×g). Consecutively, 2×10^5^ SK6-cells were infected with 1 ml of supernatant of the previous passage. This procedure was repeated every 3 to 4 days along with the determination of virus titers.

### Neutralization experiments

Virus neutralization was tested according to [49]. Briefly, serum samples from a CSFV vaccinated (S05) and a vaccinated and infected (S98) animal, as well as cell culture supernatant containing an anti-E2 antibody (A18) and a serum of an animal neither infected nor vaccinated against CSFV were diluted 2-fold in duplicates on a 96well plate (sera were kindly provided by the Community Reference Laboratory for CSF, Hannover). Thereafter, a defined virus suspension of Vp447 was added to each well and the plate was incubated for 1 h at 37°C. Subsequently, the employed virus suspension was back titrated on the plate, a suspension of SK6-cells (3×10^5^ cells/ml) was added to each well and the plates were incubated at 37°C for 72 h. Virus infection was detected by immunohistochemistry as described above. TCID_50_/ml of the employed virus suspension and ND_50_/ml were calculated according to [49].

### Immunoblotting

Western blotting was done essentially as described by (8). Briefly, 24 h–72 h after electroporation, cells were lysed in Tris-EDTA buffer containing 2% SDS, subjected to SDS-PAGE on 7.5, 10 or 12% polyacrylamide gels using Tris-tricine buffers, and blotted to nitrocellulose. As primary antibody, mouse monoclonal antibody A18 (anti-E2), 5H4 (anti-Core), 24/16 (anti-E^rns^), code 4 (anti-NS3), 6B2 (anti-NS5B) or anti-β-actin antibody (A5441; Sigma-Aldrich) was utilized. Horseradish peroxidase-coupled goat anti-mouse antibody served as secondary antibody (Dianova, Hamburg, Germany). Signals were revealed using chemiluminescence (ThermoFisher, Bonn, Germany) and exposure to Kodak BioMax film.

Virus-containing supernatants were concentrated for immunoblotting by clarification for 5 min at 3,000×g, followed by pelleting of 1.2 ml in a TL100 Beckmann ultracentrifuge at 45,000 rpm for 1 h. After removal of the supernatant, the pellet was resuspended in 10 µl Tris-EDTA buffer containing 2% SDS and further processed as described for the cell lysate. Signals were quantified employing ImageJ (http://rsbweb.nih.gov/ij/index.html).

### Sequence analysis

All constructs were confirmed by sequencing (Quiagen, Hilden, Germany). Revertant viruses were analyzed by sequencing after reverse transcriptase (RT)-PCR and cloning into the pGEM-T vector (Promega, Mannheim, Germany) using standard primers (oligonucleotide sequences are available upon request).

### Density gradient centrifugation

Continuous sucrose gradients (10%–60% w/v sucrose in 50 mM Tris, pH 7.4) of 11 ml were generated with a GP250 gradient programmer in conjunction with two Pharmacia P500 pumps at a flow rate of 1 ml/min. In a volume of 400 µl, 10^6^ ffu of each Vp447 and a Vp447 with a deletion of core protein (aa 170–246 of the polyprotein) and a five alanine linker between N^pro^ C-terminus and signal peptide (Vp447_Δc+5Ala_N_2177_Y) were layered on top of the gradient and centrifuged in a Beckman SW41 rotor at 180.000 g (32.00 rpm) for 24 h. 30 fractions of 360 µl each were collected by bottom puncture and the refractive index was determined. 30 µl of each fraction were used for titration on SK6-cells and 20 µl of two fractions pooled were subjected to Western blot analysis.

Viral RNA was purified utilizing the QuiaAmp Viral RNA kit (Quiagen, Hilden, Germany) according to the manufacturer, reverse transcribed employing the Quanti Tect Reverse Transcription kit (Quiagen, Hilden Germany) with the same reverse primer (rev: CATCCCGCGTATCTCTT) and subjected to qPCR (Quanti Tect SYBR Green PCR kit, Quiagen, Hilden, Germany) in a StepOnePlus real-time PCR system (Applied Biosystems, Darmstadt, Germany), using forward primer specific for either Vp447 (for_wt: CAAGCCACCAGAGTCCAG; fragment size 258 nt) or Vp447_Δc+5Ala_N_2177_Y (for_Δc: TGCGGCCGCAGCTCTAGA; fragment size 246 nt) and the reverse primer already employed in the reverse transcription reaction.

### Size exclusion chromatography

1×10^8^ ffu of each Vp447 and Vp447_Δc+5Ala_N_2177_Y were pelleted at 100,000×g for 1 h in a 45Ti rotor in a Beckman L8–70 ultracentrifuge. The pellet was resuspended in 550 µl 1xTNE buffer overnight at 4°C on a shaker. The complete volume was loaded onto a Pharmacia XK16 gel chromatography column, packed with Superose 6 (prep grade, GE Healthcare, Munich, Germany) with a total volume of 138 ml (determined by dextran-blue) including the void volume of 41.5 ml (determined by 10% acetone in H_2_O and subsequent measurement of optical density at 280 nm). The column was calibrated employing IgM (size 21 nm), which was subsequently measured in the elution fractions by agar gel diffusion (Novartis, Marburg, Germany). The chromatography was performed at a flow rate of 6 ml/h generated by a LKB P-1 pump with 1xTNE buffer. 80 fractions of 2 ml each were collected by a LKB superfrac collector. Collector tubes were blocked with 1xTNE containing 1% BSA fraction 5 for 10 min at room temperature. RNA was prepared from the resulting fractions by QuiaAmp Viral RNA kit (Quiagen, Hilden, Germany) and analyzed for the presence of viral genome by above described real-time RT PCR for the presence of either Vp447 or Vp447_Δc+5Ala_N_2177_Y genome.

### Transmission electron microscopy

SK6 cells transfected with either Vp447 or Vp447_Δc_N_2177_Y genome were seeded on 10 143 cm^2^ cell culture plates each in medium containing FCS. 18 h after transfection, the cells were washed twice with PBS and the medium was replaced by a serum free medium for MDBK cells (Sigma-Aldrich, Munich, Germany). 48 h after transfection, the supernatant was harvested and cellular debris was removed by centrifugation (5 min at 3,000×g). Subsequently, virus was pelleted at 25.000 rpm in a TI45 rotor for 8 h. Thereafter, the pellet was resuspended in PBS for 12 h at 4°C. Virus preparations were mounted on glow discharged, pioloform and carbon coated copper-rhodium grids. After saturation using 1% (w/v) bovine serum albumin (BSA) in PBS grids were transferred to droplets of the first antibody: monospecific rabbit serum anti E^rns^, 1∶200 in PBS, 0.5% (w/v) BSA for 1 h in a humid chamber. After 5 washing steps on droplets of PBS immune labeling was completed using goat anti-rabbit IgG conjugated to 10 nm colloidal gold (Plano, Wetzlar, Germany) 1∶25 in PBS, 0.5% (w/v) BSA. The preparation was finished by 5 washing steps on PBS followed by short incubation on distilled water and negative staining using 2% methylamine tungstate (Plano, Wetzlar, Germany). Air dried grids were examined in a Zeiss EM910 transmission electron microscope at 80 kV at an instrumental magnification of 31.500 and 50.000 and micrographs taken on Kodak SO-163 negative film.

### Animal experiment

Six weaner pigs were purchased from a commercial piggery and tested negative for infection with Pestiviruses by RT-PCR and serum neutralization test. The pigs were kept in two separately housed groups under high containment conditions. Two pigs of each group were either infected intramuscularly with 5×10^6^ TCID_50_ Vp447 or Vp447 with a deletion of core amino acids 170–246 (position in the polyprotein) (Vp447_Δc_N_2177_Y). Two days after infection, the previously separated sentinel animal was returned to each group. The animals were monitored daily for clinical signs of CSFV according to a modified clinical score developed by [27] and body temperature was recorded. The clinical score is calculated by scoring each parameter (liveliness/body tension/body shape/breathing/walking/skin/eyes+conjunctiva/appetite/defecation) from 0–3 (no signs of disease – severe signs of disease), followed by addition of all values obtained. As the animals were housed in groups in this experiment, the parameter “leftovers in feeding trough” could not be evaluated for an individual animal. EDTA blood samples were taken on days 2, 4, 7, 14, 21 and 28 after infection. The leukocyte fraction was isolated from EDTA blood by addition of 6.25% (v/v) 5% EDTA-Dextran solution, followed by sedimentation and several wash steps with PBS [49] and the leukocyte count was determined in a Neubauer chamber. Animals were euthanized because of animal welfare reasons (clinical score >20 or severe disease) during the experiment or at the end of the experiment.

## Supporting Information

Figure S1 **Western blot analysis employing antibodies directed against CSFV E^rns^, E2, NS3 and NS5B of SK6-cells transfected with genomes of Vp447_Δc_, Vp447_Δc_N_2177_Y and Vp447.** Cells were lysed 72 h after transfection and the lysate was separated on 7.5% tricine gels. Mock transfected cells serve as negative control ( = neg). E^rns^ was detected by mouse mab 24/16, E2 by A18, NS3 by code 4 and NS5B by 6D2. Detection of β-actin was performed to compare the amount of cell lysate loaded onto the gel.
(TIF)

Figure S2 **Specificity of qPCRs amplifying either Vp447 (wt) or Vp447_Δc+5Ala_N_2177_Y (Δc) genomes.** Specificity of virus specific real-time RT-PCRs depicted as Ct-value per given amount of cDNA plasmid. wt = Vp447; Δc = Vp447_Δc_N_2177_Y.
(TIF)

Figure S3 **Comparison of E2-, RNA- and infectivity distribution according to density in the supernatant of Vp447_Δc+5Ala_N_2177_Y and Vp447_Δc+5Ala_N_2177_ genome transfected cells.** 75 ml each of supernatant of Vp447_Δc+5Ala_N_2177_Y and Vp447_Δc+5Ala_N_2177_ genome transfected SK6 cells was harvested 48 h after transfection. The supernatant was concentrated by ultracentrifugation and subsequently subjected to equilibrium density centrifugation. (A) Infectivity and RNA-content, as well as (B) E2-levels were determined according to density. The relative E2 signal in percent compared to the total E2 signal is indicated below the blots. ΔcN_2177_Y = Vp447_Δc+5Ala_N_2177_Y; ΔcN_2177_ = Vp447_Δc+5Ala_N_2177_.
(TIF)

Figure S4 **Comparison of E2, infectivity and RNA distribution of Vp447 (wt) versus Vp447_H30K (H30K).** Both viruses were subjected to equilibrium centrifugation, with or without prior treatment with 0.5% NP40. Thereafter, (A) infectivity and RNA levels were determined according to density, as was (B) the distribution of E2.
(TIF)

Figure S5 **Comparison of E2, infectivity and RNA distribution of Vp447_Δc+5Ala_N_2177_Y (Δc) versus Vp447_Δc+5Ala_N_2177_Y_H30K (ΔcH30K).** Both viruses were subjected to equilibrium centrifugation, with or without prior treatment with 0.5% NP40. Thereafter, infectivity and RNA levels were determined according to density.
(TIF)

Figure S6 **Specificity of serum used in EM.** Pictures show negative control (cell culture supernatant treated like virus preparation) {neg} and preparation of Vp447 {wt} at a magnification of ×31,500 which were stained as described in Materials & Methods.
(TIF)

Figure S7 **Thermostability of Vp447 and Vp447_Δc_N2177Y.** Defined virus preparations of Vp447 and Vp447_Δc_N2177Y were incubated for 2, 4, 12, 36 and 48 h at 37°C (A) and 39.5°C (B) and virus titer was determined in ffu/ml. (C) Virus particles were subjected to one cycle of freezing thawing and virus titer was determined in ffu/ml before and afterwards. Depicted are mean and standard deviation of n = 3 experiments. wt = Vp447; Δc = Vp447_Δc_N2177Y.
(TIF)

Figure S8 **Subdomain organization of NS3 and localization of single amino acid substitutions within NS3 helicase.** CSFV NS3 helicase subdomain 3 is presented as multiple sequence alignment (ClustalW) with HCV, GBV-A, GBV-C and dengue virus 4 (DV4). Residues of single amino acid substitutions are underlined, substituted amino acids and position in the polyprotein are written above the respective residues. Grey background represents α-helices with reference to structures by Luo et al. (2008) and Appleby et al. (2011). Accession: HCV: gi: 316983284; GBV-A: gi: 9629719; GBV-C: gi: 9628706; DV4: gi: 159795581.
(TIF)
