# Supplementary figures and images for: The Core Protein of Classical Swine Fever Virus Is Dispensable for Virus Propagation In Vitro
Source: PLoS Pathog. 2012 Mar 22;8(3):e1002598. doi: 10.1371/journal.ppat.1002598 (PMC3310793; doi:10.1371/journal.ppat.1002598)

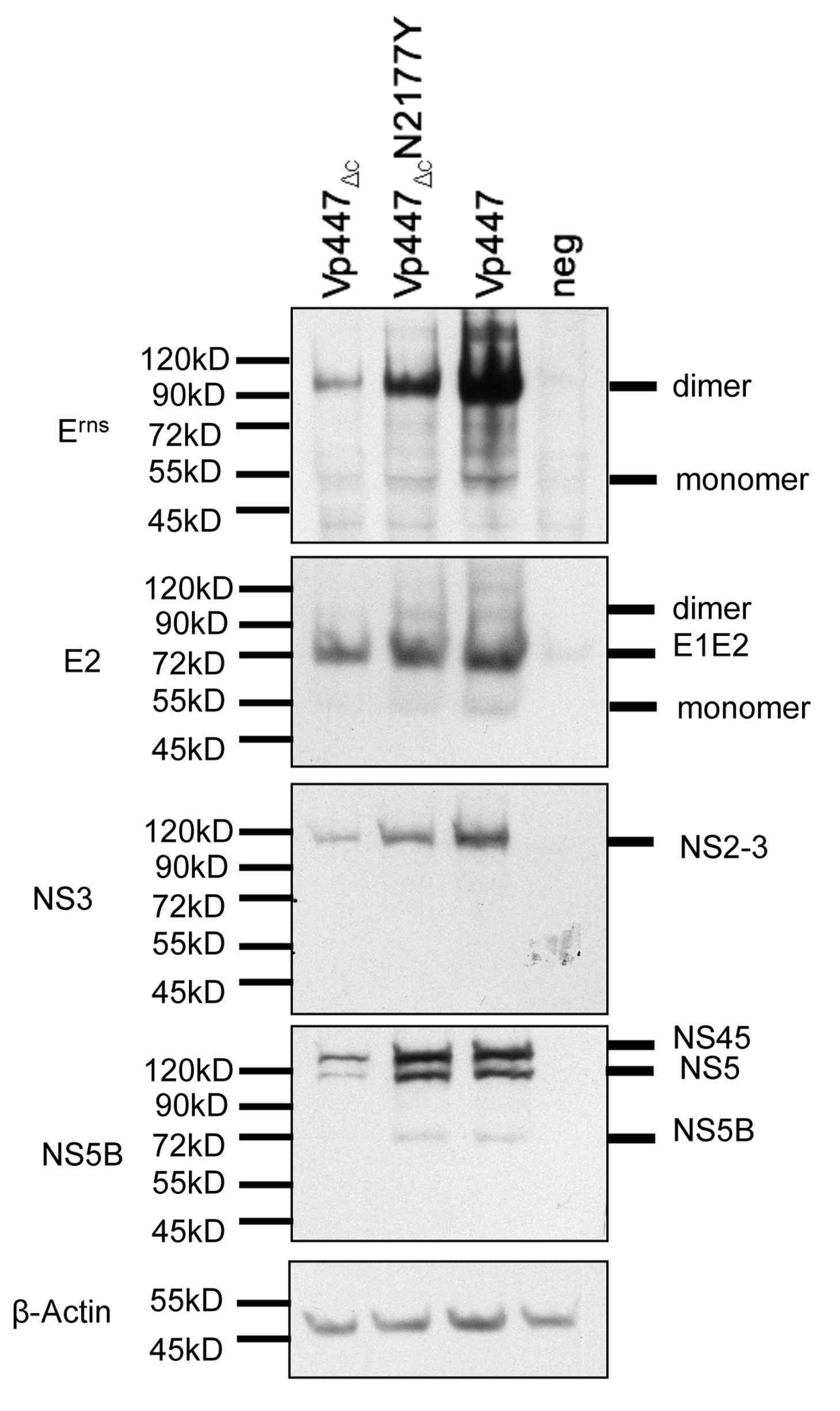

Supplement: Figure S1 — Western blot analysis employing antibodies directed against CSFV Erns, E2, NS3 and NS5B of SK6-cells transfected with genomes of Vp447Δc, Vp447ΔcN2177Y and Vp447. Cells were lysed 72 h after transfection and the lysate was separated on 7.5% tricine gels. Mock transfected cells serve as negative control ( = neg). Erns was detected by mouse mab 24/16, E2 by A18, NS3 by code 4 and NS5B by 6D2. Detection of β-actin was performed to compare the amount of cell lysate loaded onto the gel. (TIF) [file ppat.1002598.s001.tif]

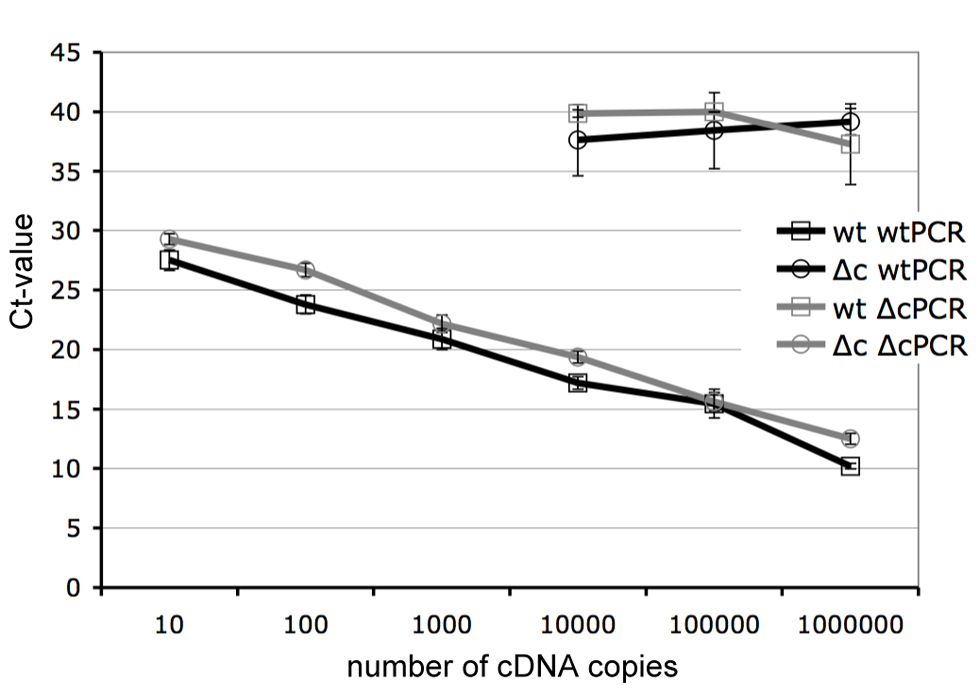

Supplement: Figure S2 — Specificity of qPCRs amplifying either Vp447 (wt) or Vp447Δc+5AlaN2177Y (Δc) genomes. Specificity of virus specific real-time RT-PCRs depicted as Ct-value per given amount of cDNA plasmid. wt = Vp447; Δc = Vp447ΔcN2177Y. (TIF) [file ppat.1002598.s002.tif]

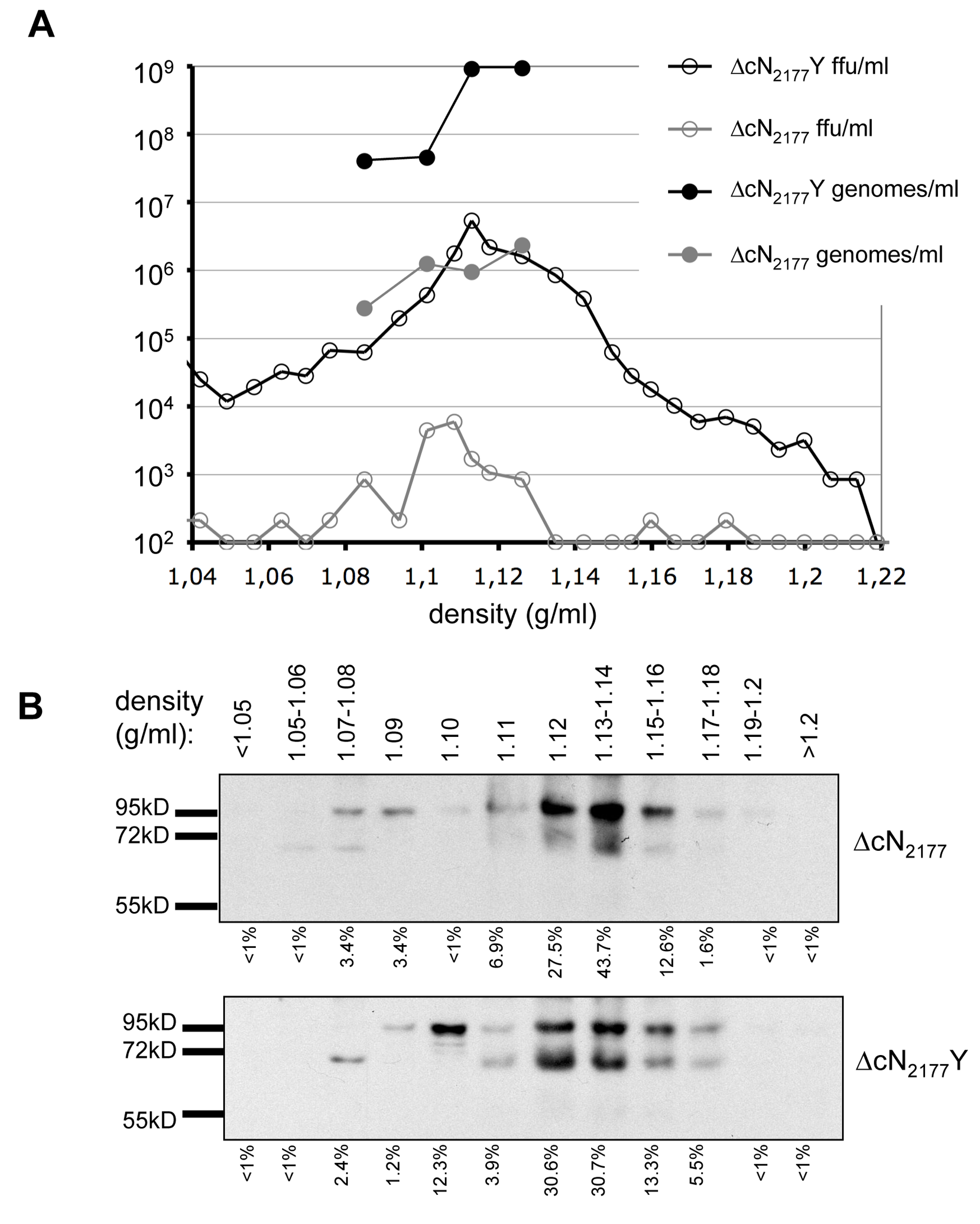

Supplement: Figure S3 — Comparison of E2-, RNA- and infectivity distribution according to density in the supernatant of Vp447Δc+5AlaN2177Y and Vp447Δc+5AlaN2177 genome transfected cells. 75 ml each of supernatant of Vp447Δc+5AlaN2177Y and Vp447Δc+5AlaN2177 genome transfected SK6 cells was harvested 48 h after transfection. The supernatant was concentrated by ultracentrifugation and subsequently subjected to equilibrium density centrifugation. (A) Infectivity and RNA-content, as well as (B) E2-levels were determined according to density. The relative E2 signal in percent compared to the total E2 signal is indicated below the blots. ΔcN2177Y = Vp447Δc+5AlaN2177Y; ΔcN2177 = Vp447Δc+5AlaN2177. (TIF) [file ppat.1002598.s003.tif]

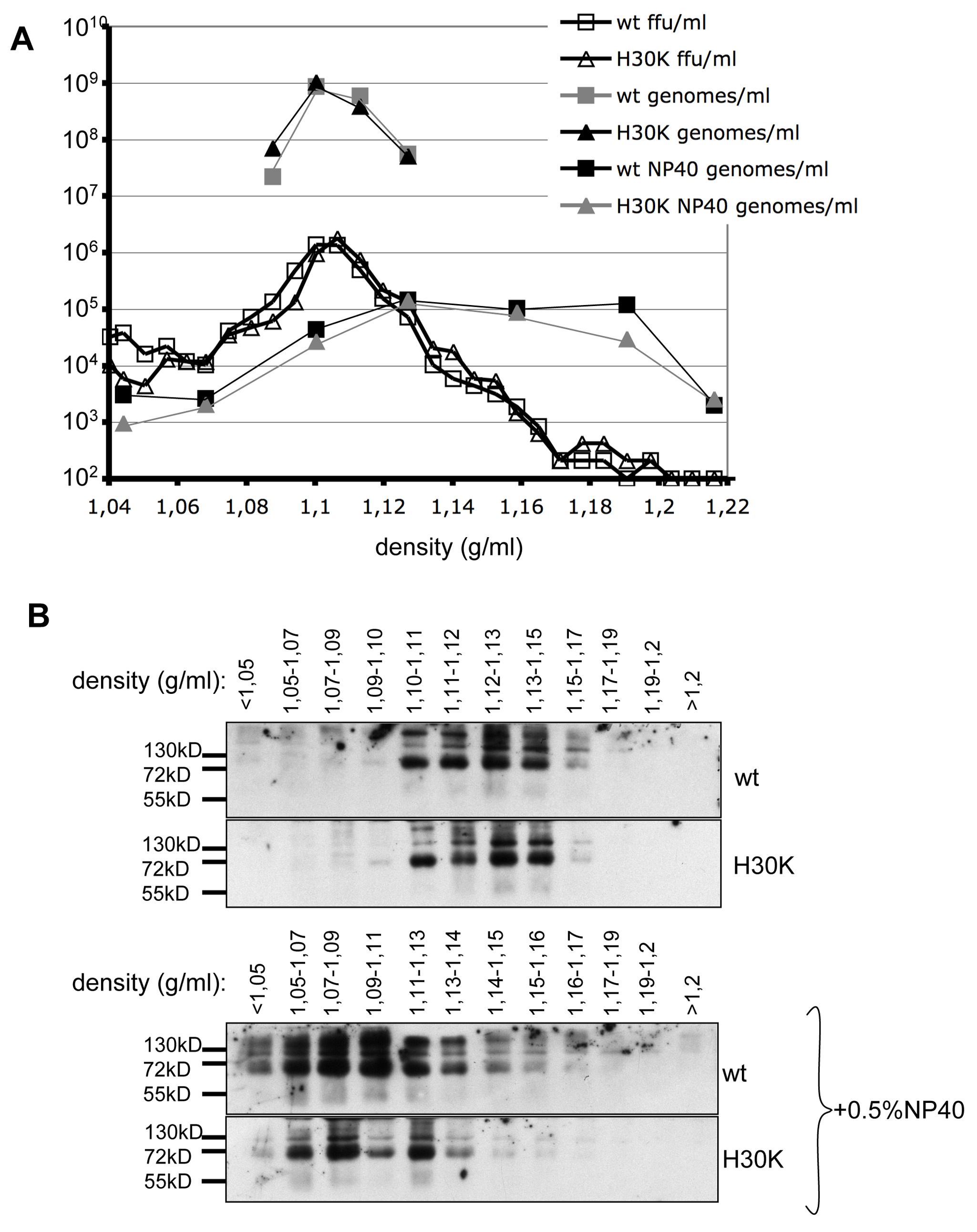

Supplement: Figure S4 — Comparison of E2, infectivity and RNA distribution of Vp447 (wt) versus Vp447_H30K (H30K). Both viruses were subjected to equilibrium centrifugation, with or without prior treatment with 0.5% NP40. Thereafter, (A) infectivity and RNA levels were determined according to density, as was (B) the distribution of E2. (TIF) [file ppat.1002598.s004.tif]

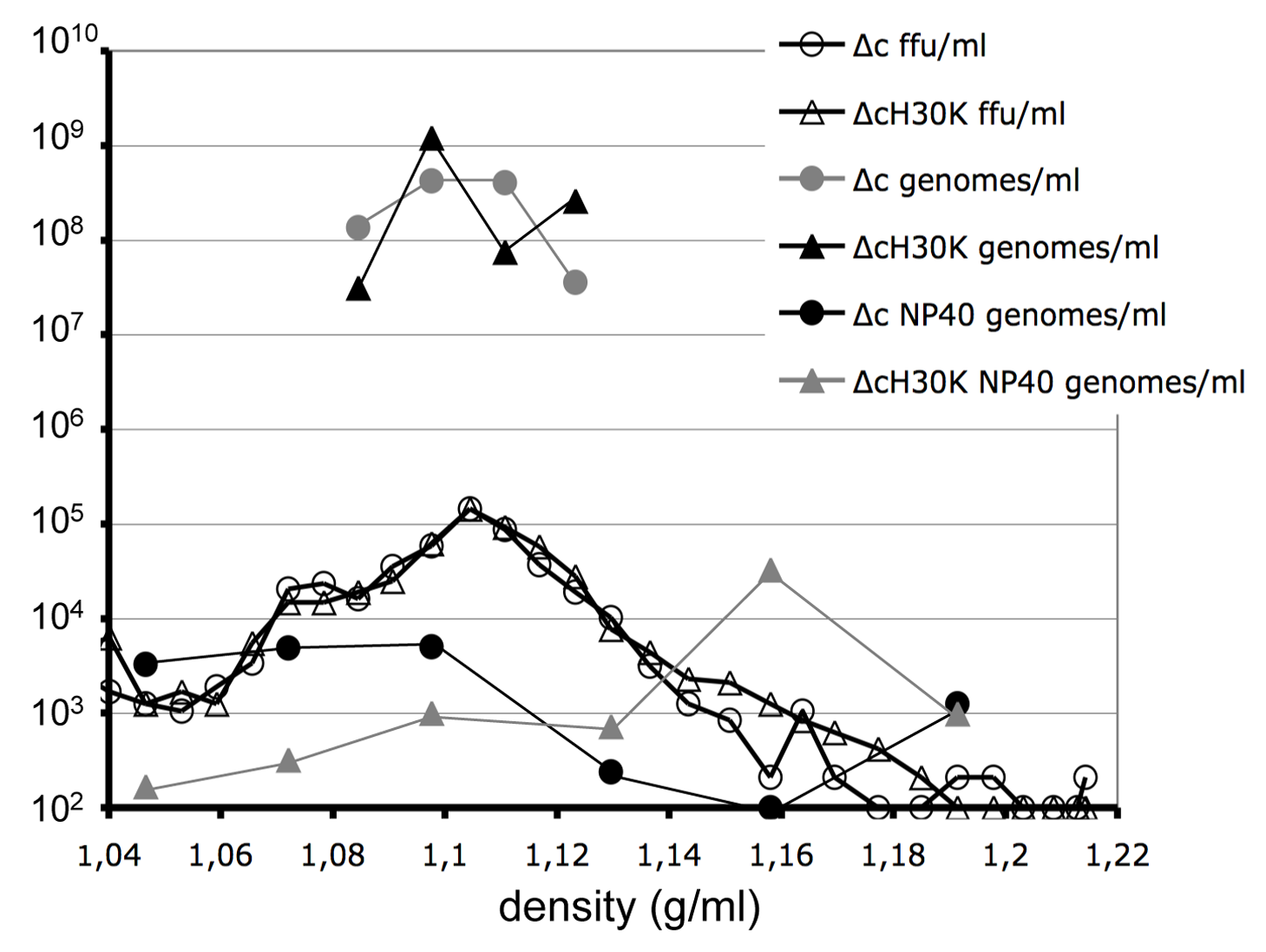

Supplement: Figure S5 — Comparison of E2, infectivity and RNA distribution of Vp447Δc+5AlaN2177Y (Δc) versus Vp447Δc+5AlaN2177Y_H30K (ΔcH30K). Both viruses were subjected to equilibrium centrifugation, with or without prior treatment with 0.5% NP40. Thereafter, infectivity and RNA levels were determined according to density. (TIF) [file ppat.1002598.s005.tif]

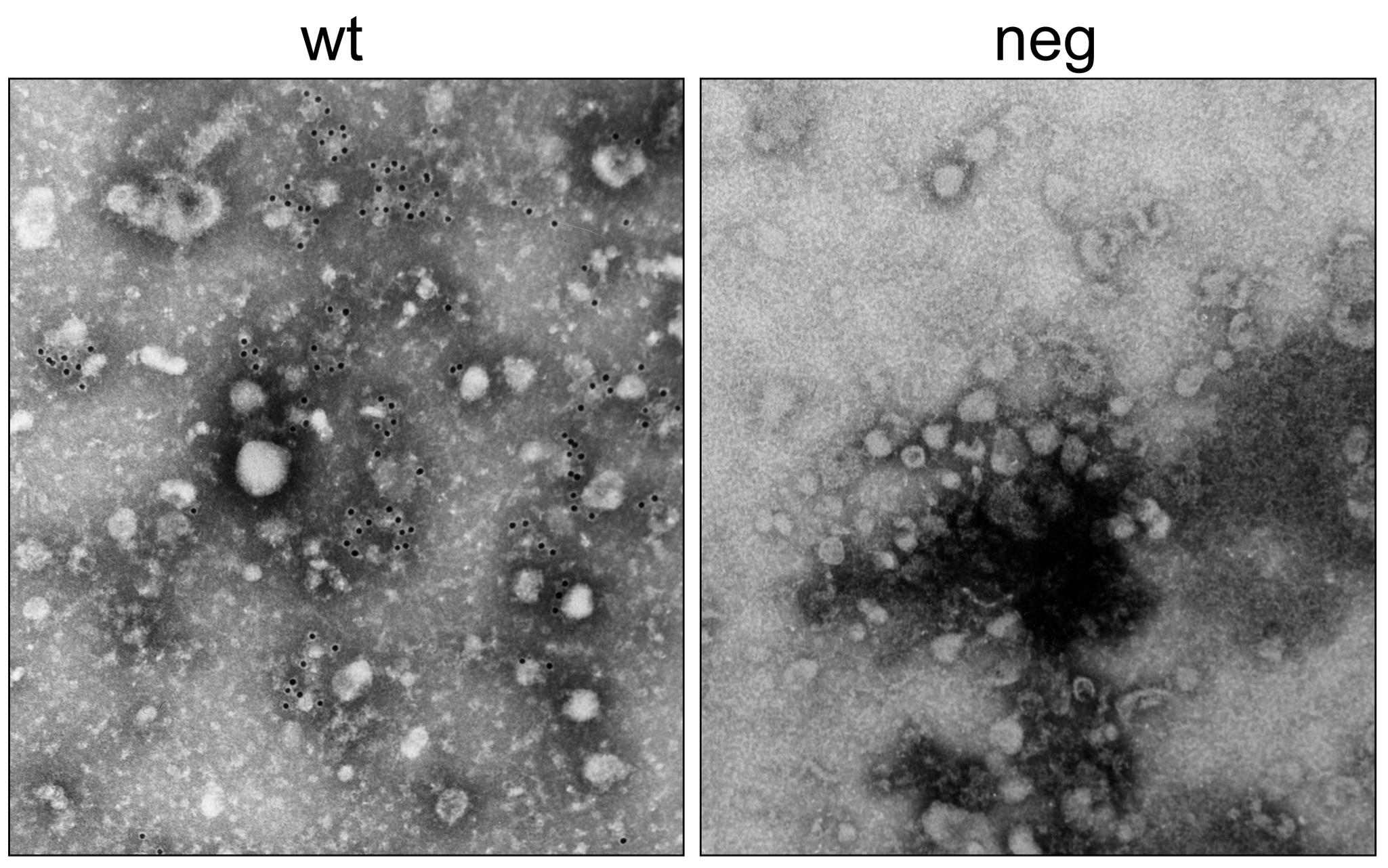

Supplement: Figure S6 — Specificity of serum used in EM. Pictures show negative control (cell culture supernatant treated like virus preparation) {neg} and preparation of Vp447 {wt} at a magnification of ×31,500 which were stained as described in Materials & Methods. (TIF) [file ppat.1002598.s006.tif]

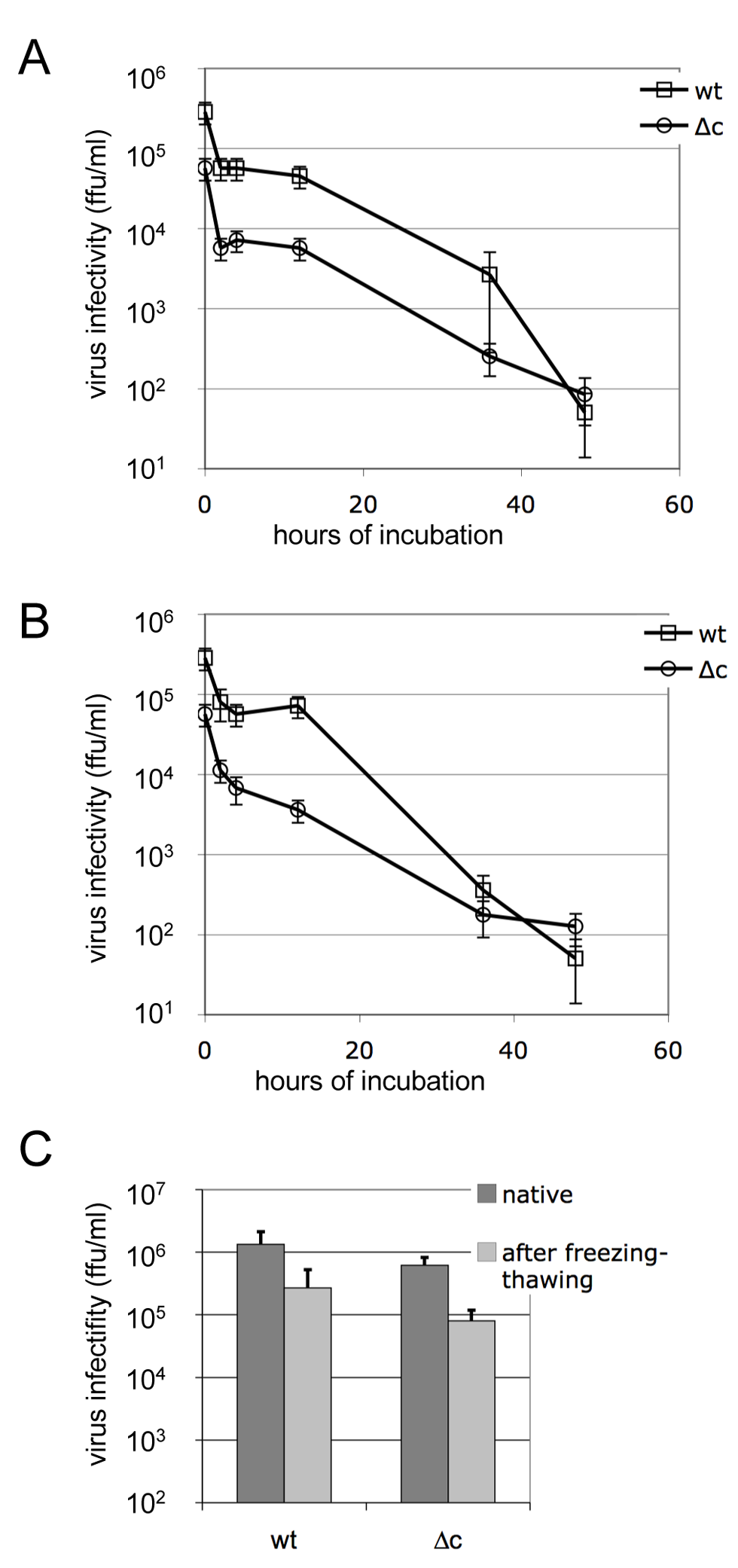

Supplement: Figure S7 — Thermostability of Vp447 and Vp447ΔcN2177Y. Defined virus preparations of Vp447 and Vp447ΔcN2177Y were incubated for 2, 4, 12, 36 and 48 h at 37°C (A) and 39.5°C (B) and virus titer was determined in ffu/ml. (C) Virus particles were subjected to one cycle of freezing thawing and virus titer was determined in ffu/ml before and afterwards. Depicted are mean and standard deviation of n = 3 experiments. wt = Vp447; Δc = Vp447ΔcN2177Y. (TIF) [file ppat.1002598.s007.tif]

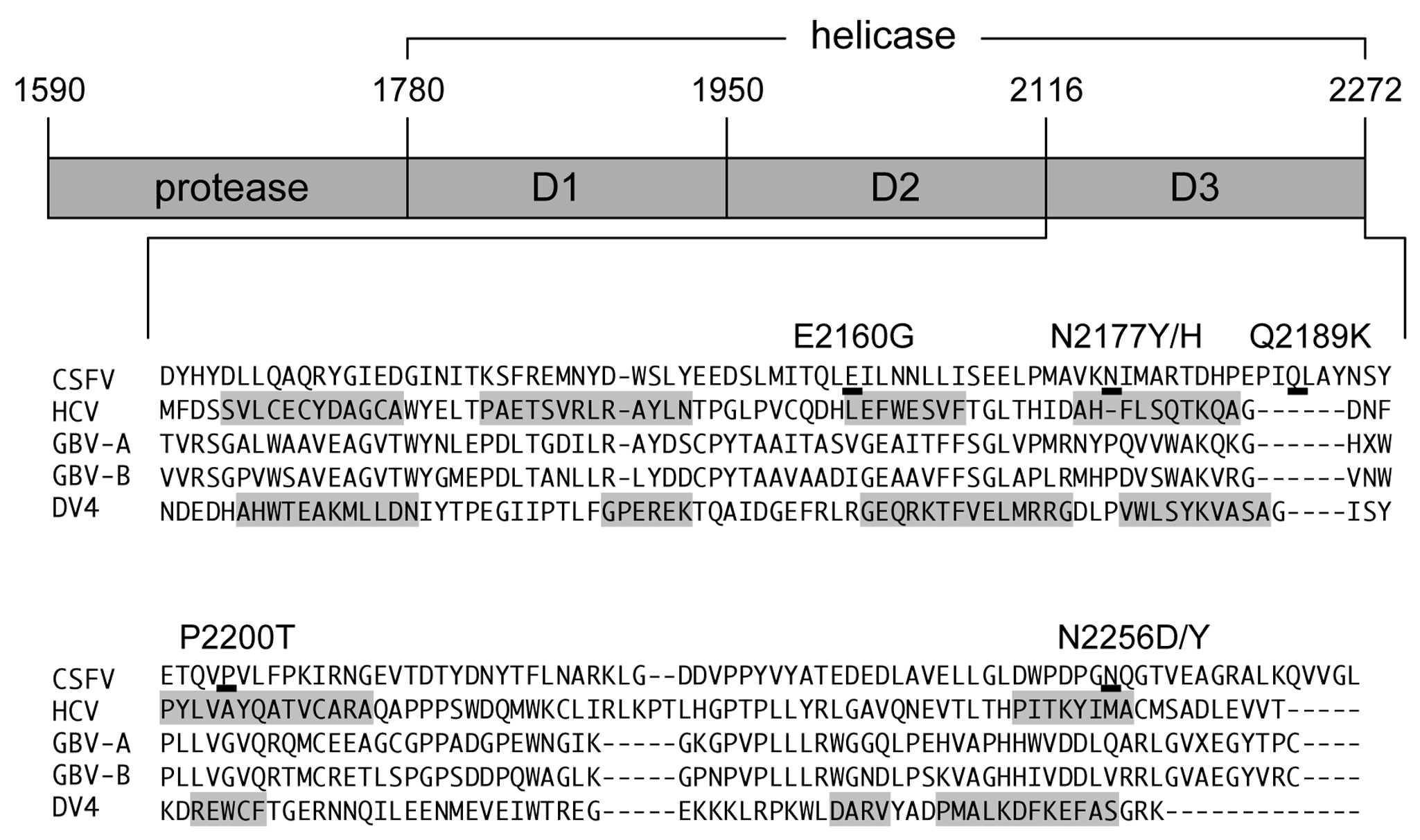

Supplement: Figure S8 — Subdomain organization of NS3 and localization of single amino acid substitutions within NS3 helicase. CSFV NS3 helicase subdomain 3 is presented as multiple sequence alignment (ClustalW) with HCV, GBV-A, GBV-C and dengue virus 4 (DV4). Residues of single amino acid substitutions are underlined, substituted amino acids and position in the polyprotein are written above the respective residues. Grey background represents α-helices with reference to structures by Luo et al. (2008) and Appleby et al. (2011). Accession: HCV: gi: 316983284; GBV-A: gi: 9629719; GBV-C: gi: 9628706; DV4: gi: 159795581. (TIF) [file ppat.1002598.s008.tif]
